# Calibrated Deep-Learning Risk Indexing and Latent Behavioural Profiling for Occupational Mental-Health Risk Assessment

**DOI:** 10.3390/bioengineering13060626

**Published:** 2026-05-27

**Authors:** Abuzar Khan, Khalid Rehman, Ahmad Junaid, Abid Iqbal, Muhammad Farooq Siddique, Muhammad Ismail Mohmand, Ghassan Husnain

**Affiliations:** 1 Department of Computer Science, CECOS University of IT and Emerging Sciences, Peshawar 25100, Pakistan; abuzarkhaan580@gmail.com (A.K.); ahmadjunaid@cecos.edu.pk (A.J.); ghassan.husnain@gmail.com (G.H.); 2 Department of Electrical Engineering, CECOS University of IT and Emerging Sciences, Peshawar 25100, Pakistan; khalid@cecos.edu.pk; 3 Department of Computer Engineering, College of Computer Sciences and IT, King Faisal University, Al Ahsa 31982, Saudi Arabia; aaiqbal@kfu.edu.sa; 4 Department of Mechanical Engineering, University of Engineering and Technology, Mardan 23200, Pakistan; farooqsiddique.mech@gmail.com; 5 Department of Computer Engineering, Faculty of Engineering and Natural Sciences, Istanbul Atlas University, Istanbul 34408, Turkey

**Keywords:** computational intelligence for healthcare, public mental health, occupational mental health, psychosocial support, workplace survey data, knowledge work, risk index, latent profiling, health disparities, ethical AI, transfer learning

## Abstract

Occupational mental-health risk in knowledge-work settings is an important public-health and psychosocial-support concern because workload demands, career insecurity, limited mentoring, uneven institutional support and barriers to care can increase psychological risk, including in early-career academic environments. Workplace well-being assessments rely on aggregate survey summaries or conventional prediction models, limiting calibration, interpretability, subgroup evaluation and transfer validation. This study develops a computational-intelligence framework for public mental-health decision support using heterogeneous workplace survey data with early-career academics treated as a motivating knowledge-work context rather than as the direct empirical cohort. The proposed approach combines attention-based tabular learning, variational autoencoder latent profiling, stacked ensemble prediction, probability calibration, feature attribution, perturbation analysis, fairness assessment and cross-dataset adaptation. Calibrated probabilities are converted into a transparent 0–100 risk index to support preventive outreach, psychosocial-support planning and resource-allocation decisions. The model is compared with baselines, including logistic regression, support vector machine, random forest, XGBoost, LightGBM, CatBoost, TabNet, FT–Transformer, NODE and DCN. Results show strong held-out performance with AUC = 0.885, average precision = 0.872, F1 = 0.808, Brier score = 0.145 and expected calibration error = 0.022, outperforming tested baselines. Five-fold robustness analysis produced a conservative mean test AUC of 0.809±0.044, indicating moderate partition sensitivity. Key predictors include work interference, perceived stress, care access and support variables. Latent profiling identifies two behavioural subgroups with distinct risk patterns. After feature harmonization, target-domain adaptation and recalibration, external evaluation on an occupational burnout dataset achieves AUC = 0.941 and average precision = 0.936, supporting calibrated, interpretable and subgroup-aware decision support under dataset shift.

## 1. Introduction

### 1.1. Occupational Stress as a Data-Intensive Public Health Problem

**Figure 1:**
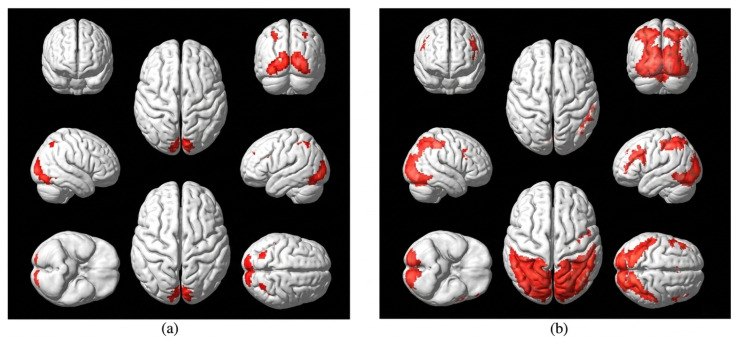
Conceptual illustration of stress-related brain activation differences between (**a**) moderate-stress and (**b**) high-stress conditions. The red-colored regions indicate brain areas showing stress-related activation differences, with more extensive red regions representing higher activation differences under the high-stress condition. Adapted from neuroimaging findings by Choi and Choi [4].

Occupational stress has become a major determinant of mental health and workforce sustainability and institutional performance within knowledge-intensive environments [1]. This problem becomes more important in high-pressure professional settings because productivity expectations, role ambiguity, job insecurity, social support and access to care jointly shape psychological risk. Academic work provides a strong example of knowledge-work stress because professional identity, scientific productivity, teaching responsibility, administrative demand and career uncertainty remain tightly connected [2]. Early-career researchers may face publication pressure, competitive funding environments, insecure contracts, limited autonomy and uneven mentoring access which together increase occupational vulnerability [3]. These pressures can gradually accumulate into chronic stress, emotional exhaustion, anxiety symptoms, depressive symptoms, reduced engagement and intention to leave the profession. Neuroimaging evidence further suggests that stress severity is associated with distinct brain activation patterns which are illustrated in Figure 1.

**Figure 2:**
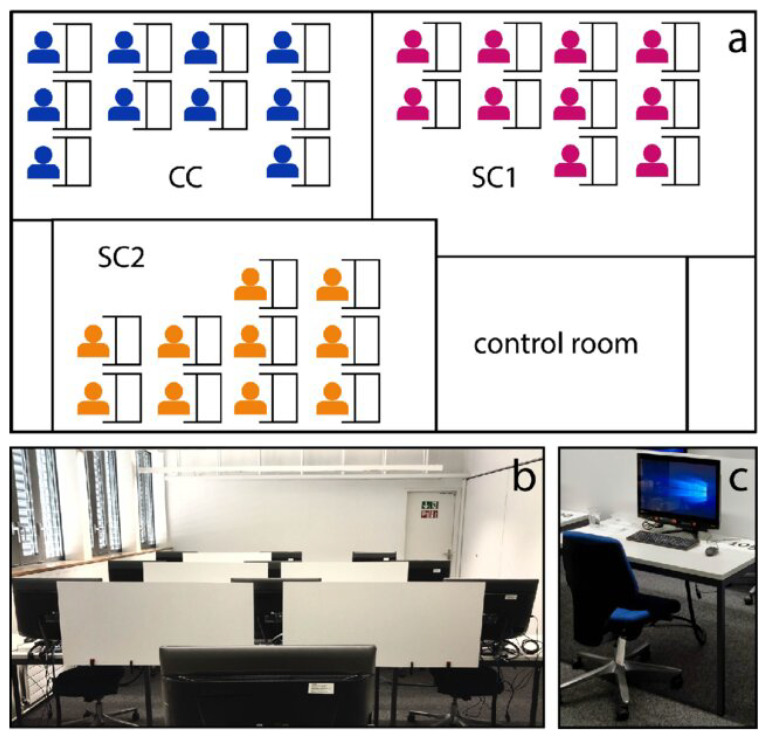
Floor plan and images of the open-office environment used in an acute work-stress experiment: (**a**) floor plan of the laboratory/open-office layout, (**b**) view of the workstation arrangement, and (**c**) view of the participant work area during the experiment. The colors in the floor plan indicate different spatial zones, workstation positions, and layout components used to distinguish areas within the experimental office environment. Adapted from Kerr et al. [11].

The mental health burden linked with occupational stress is not only an individual clinical concern because it also reflects a broader public health challenge shaped by organizational structures, resource allocation, stigma and confidentiality concerns and timely care access [5]. The workplace therefore becomes a sociotechnical setting where psychosocial exposures are produced and amplified and sometimes mitigated through institutional policies and support systems. Swarm-based coordination research also shows how complex adaptive systems can support structured evaluation and decision support under uncertain conditions [6]. An illustrative occupational-stress setting is shown in Figure 2 where acute work stress was examined within a controlled group-office environment.

High workload, limited job control, weak supervisor support, unclear career pathways and confidentiality uncertainty may reduce help-seeking behaviour while delaying timely intervention [7]. Supportive supervision, transparent policies, accessible services, flexible work arrangements and psychologically safe environments can instead reduce risk while improving resilience across vulnerable occupational groups. These patterns indicate that occupational mental health should be studied as a complex sociotechnical system rather than as isolated symptoms or individual weakness [8]. Efficient intrusion-detection research further illustrates how adaptive monitoring can support risk recognition in complex networked environments, which conceptually supports structured occupational-risk detection [9]. The COVID-19 pandemic also demonstrated the importance of healthcare data analytics for public-health decision support because data integration, predictive modelling, risk monitoring and resource planning became essential during rapidly changing healthcare conditions [10].

Traditional workplace well-being assessment often relies on periodic surveys, aggregate descriptive statistics and broad risk categories which provide useful monitoring but limited individual or subgroup insight. These approaches become insufficient when the objective is to identify heterogeneous risk profiles, detect nonlinear interactions among stressors and support targeted preventive action. Knowledge-work populations differ across occupation, organization size, country, gender, work arrangement, career stage and support availability, which means average estimates can hide important subgroup patterns [12]. Scalable computational methods are therefore needed to convert heterogeneous workplace survey signals into interpretable risk information that can support prevention, outreach and policy design while preserving the social meaning of mental-health data.

### 1.2. Computational Intelligence for Interpretable Risk Modelling

Computational intelligence provides a practical route for modelling complex psychosocial risk because it can integrate demographic, behavioural, occupational and organizational variables within a unified analytical framework. Machine learning is especially useful for tabular survey data when stress-related outcomes emerge from interacting effects rather than independent linear relationships [13]. Workload interference may have different implications depending on supervisor support, care access, remote-work status, organizational culture and previous help-seeking experience. Such dependencies require flexible representations that remain understandable to researchers and decision makers who must interpret risk patterns responsibly.

Attention-based tabular models are well suited to this problem because they learn embeddings for sparse categorical variables, capture contextual feature relationships and model higher-order interactions where feature importance varies by respondent context [14]. Sparse-attention mechanisms can further support structured tabular learning by emphasizing informative variables while reducing the influence of noisy or weakly relevant predictors [15]. Image steganalysis research also demonstrates that attention mechanisms can improve pattern recognition when important signals are subtle and distributed across complex inputs [16]. Occupational mental-health risk often emerges from cumulative stressors involving workload, stigma, care access, support, anonymity and personal history, which makes attention-based learning useful for nuanced risk stratification [17]. Attribution analysis, attention analysis, latent profiling, perturbation testing and subgroup evaluation are therefore needed to verify whether predictions reflect meaningful occupational signals rather than unstable artefacts [18].

The present work develops an interpretable deep-learning risk index for occupational mental-health modelling using heterogeneous workplace survey data and calibrated probability outputs. Calibrated probabilities are mapped onto a transparent 0–100 score so that risk outputs become easier to interpret for non-technical stakeholders and institutional decision makers. Engine-failure prediction research also demonstrates how calibrated risk-oriented modelling can support early warning and decision support in complex operational settings [19]. This formulation relates to MEGA-style healthcare risk indexing where heterogeneous signals are translated into actionable risk scores for decision-support applications [20]. Attention-guided abnormal-pattern detection provides an additional reference because it supports robust identification of high-risk patterns under complex input conditions [21].

The proposed framework is methodologically aligned with topology-aware temporal modelling because both approaches seek to capture structured dependencies among complex features [22]. Temporal network prediction provides additional conceptual support because evolving relationships can improve predictive structure within dynamic systems [23]. Early abnormal-pattern identification in public-health settings further supports preventive modelling where risk is detected before outcomes become severe [24]. Unsupervised anomaly detection also motivates latent high-risk pattern discovery because important profiles may not be fully captured by predefined labels [25]. Explainable vision-transformer research further reinforces the need to connect deep representation learning with interpretable outputs for high-stakes decision contexts [26].

Recent deep-learning studies also support the broader design logic of the proposed occupational risk framework. Multiscale feature learning shows how complex signals can be represented across several levels of abstraction [27]. Convolution-based representation extraction remains useful when local patterns carry predictive information within structured input spaces [28]. Wireless vehicle modelling further demonstrates that learned representations can generalize across diverse signal environments and predictive domains [29]. Hybrid CNN–Transformer integration is relevant because it combines local feature extraction with long-range dependency modelling for complex sequence and pattern tasks [30]. Dense-sparse coding shows how compact feature representations can improve downstream prediction while reducing irrelevant variation [31]. Difficulty-aware fine-grained prediction is useful when observations vary in ambiguity and classification difficulty across heterogeneous cases [32]. Adaptive cross-channel dependency learning also motivates treating survey variables as interacting signals rather than independent predictors [33].

Early-career academics motivate the study, while the empirical task remains broader with occupational risk prediction from workplace survey signals across mental-health and burnout datasets. The proposed framework combines attention-enhanced prediction, latent representation learning, ensemble calibration and explanation for preventive decision support [34]. This decision-support perspective relates to social-feedback-aware behavioural modelling in online health contexts where behavioural signals help explain professional participation and user response patterns [35]. It also connects with online fitness socializing research where social interaction data are used to interpret health-related behaviour and engagement patterns [36]. Knowledge-enhanced cross-modal medical representation learning provides another methodological reference because it integrates heterogeneous health-related signals into richer predictive representations [37].

### 1.3. Study Rationale and Contributions

This study is motivated by the need to connect predictive analytics, psychosocial assessment and institutional decision making within occupational mental health. Existing well-being initiatives often respond after distress has become severe, while conventional survey summaries may lack the granularity required for early prevention. A computational risk framework can help identify elevated occupational stress, distinguish lower-risk and higher-risk profiles and highlight organizational conditions associated with increased risk. Such a framework is particularly relevant to knowledge-intensive populations including early-career academics, while empirical validation in this study uses broader workplace mental-health and burnout datasets.

**Figure 3:**
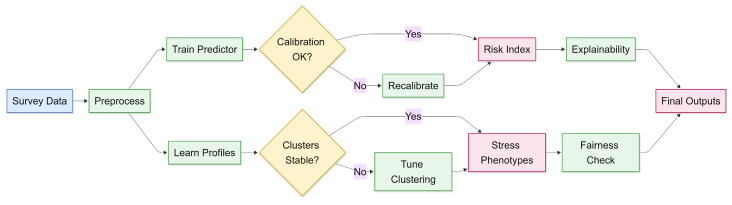
Overall study flow of the proposed occupational mental-health risk-modelling framework showing preprocessing, calibrated prediction, stress-phenotype discovery, explainability, fairness assessment and final decision-support outputs.

This study therefore makes three connected contributions that address prediction and profiling and validation together. First the study develops a calibrated risk-prediction pipeline using heterogeneous workplace survey features that represent demographic, occupational, behavioural and support-related conditions. Second the study applies representation learning and clustering to identify latent occupational-stress profiles characterized by differences in support, work interference, stress burden and care access. Third the study evaluates robustness through cross-validation, perturbation analysis, subgroup fairness assessment and transfer testing across occupational mental-health datasets. The overall study flow is summarized in Figure 3 where survey data are preprocessed and passed through two complementary branches for calibrated risk prediction and latent-profile discovery. The resulting risk index, occupational-stress profiles, explainability outputs and fairness checks provide a transparent basis for preventive decision support.

The remainder of this paper is organized so that Section 2 reviews relevant studies and gaps, while Section 3 describes the proposed framework, Section 4 presents the results, Section 5 discusses implications and comparisons and Section 6 concludes with limitations and future work.

## 2. Related Work

### 2.1. Computational Approaches to Occupational and Public Mental Health

Mental health research has increasingly adopted computational methods to analyse behavioural, clinical, social and organizational data for risk understanding and decision support [38]. This shift from retrospective description toward anticipatory analytics is particularly relevant because psychological risk often develops gradually through interacting social, occupational and behavioural factors. Occupational mental-health studies consistently identify workload, job insecurity, low control, stigma, poor managerial support and limited care access as recurring determinants of distress. Academic work shares these risks while also including pressures related to publication expectations, grant competition, disciplinary reputation, mobility and precarious career progression [39].

Early-career researchers often face high performance expectations while having limited influence over institutional policies which can intensify stress and reduce help-seeking when confidentiality or career consequences are major concerns [40]. Smart predictive-maintenance research also illustrates how early-warning logic can support timely intervention before system-level deterioration becomes severe [41]. These findings make early-career academics an important motivating context for occupational mental-health decision support even when model development uses broader workplace survey data. Many applied studies rely on regression models, descriptive comparisons and conventional machine-learning methods such as random forests, gradient boosting and support vector machines. Multiclass support-vector classification with active learning also provides a related example of robust classification under complex feature conditions [42]. As multi-source health and behavioural data become more available, computational intelligence can uncover patterns that are difficult to detect manually, while these models must remain interpretable, clinically cautious, fairness-aware and aligned with preventive institutional uses [43]. Secure federated-learning research further reinforces the importance of privacy-preserving analytics when sensitive health-related data are used for predictive modelling [44].

### 2.2. Deep Learning for Heterogeneous Tabular Health Data

Deep learning has advanced imaging, biosignal analysis, natural language processing and multimodal health analytics, while its application to tabular health data remains more challenging because such data include mixed variable types, missingness, high-cardinality categorical fields, modest sample sizes and complex feature dependencies [45]. Transformer-based biomedical research also shows how attention mechanisms can support flexible representation learning across heterogeneous health-related tasks [46]. Recent tabular neural architectures including attention-based models, masked feature-selection networks, differentiable decision structures and hybrid ensembles have improved modelling of heterogeneous structured data where interactions among variables are central [47]. Attention mechanisms are particularly useful for survey-based mental-health modelling because they allow feature importance to vary across respondents and contexts. This capability is consistent with review evidence showing that attention-based deep-learning frameworks can support adaptive feature weighting in complex physiological and behavioural modelling tasks [48]. EEG fatigue-review evidence also supports the broader relevance of attention-guided learning for modelling complex human-state signals [49]. Entropy-informed residual modelling further demonstrates that nonlinear human-state dynamics can be captured through deep architectures when physiological variation is complex [50].

In occupational stress analysis the same exposure may carry different implications depending on supervisory support, anonymity, benefits, remote-work status and previous mental-health history [51]. Attention-based tabular learning therefore provides a flexible mechanism for capturing respondent-specific feature relevance and conditional dependencies that conventional models may miss [52]. Representation learning further supports latent subgroup discovery because compressed embeddings can preserve behavioural and organizational structure in lower-dimensional spaces. Generative augmentation can support imbalanced tabular learning although synthetic data must be evaluated carefully because poor reconstruction of continuous variables or joint dependencies can distort risk patterns [53]. Recent deep-learning studies further show that recurrent architectures, hybrid CNN-DNN mechanisms, stacked ensembles and multihead feature-extraction frameworks can strengthen representation learning and classification in complex pattern-recognition tasks.

Bidirectional recurrent modelling demonstrates how sequential dependencies can improve prediction when temporal structure is informative [54]. Hybrid Android-detection research illustrates the value of combining complementary neural components for improved classification under complex feature conditions [55]. Stacked ensemble malware-detection work provides evidence that layered predictive systems can improve robustness across challenging classification settings [56]. Multihead feature-extraction frameworks also support the idea that different representation channels can capture complementary aspects of heterogeneous input data [57].

### 2.3. Interpretability, Calibration, Fairness and Transferability

Responsible use of computational intelligence in mental health requires more than strong classification accuracy because model outputs may influence support allocation and institutional decision making [58]. Interpretability methods such as SHAP, integrated gradients, feature perturbation and attention analysis help identify whether predictions are driven by plausible psychosocial and organizational factors. Calibration is equally important because predicted probabilities should correspond to observed outcome frequencies before being converted into decision thresholds or operational risk bands [59]. Uncertainty-aware active learning further supports the need to quantify uncertainty when predictive systems are deployed in complex decision-support settings [60]. Improved KNN-based classification also demonstrates the value of robust baseline learning when high-stakes classification tasks require stable decision boundaries [61]. Adaptive swarm optimization provides another reference for improving search and optimization under complex operational constraints [62]. False-alarm reduction research further supports careful threshold design when decision-support systems must avoid unnecessary alerts and missed risks [63].

Fairness and transferability remain central challenges because subgroup differences in culture, care access, help-seeking, reporting behaviour, gender, employment context and region can produce unequal error patterns that aggregate metrics conceal [64]. Subgroup AUC, true-positive-rate differences, true-negative-rate differences and calibration gaps are therefore needed to evaluate whether risk estimates behave equitably across worker groups [65]. Models trained in one institutional or regional setting may also fail under domain shift, which makes local validation, transfer testing and fine-tuning necessary before practical deployment [66]. These gaps motivate the present framework because it integrates calibrated risk indexing, interpretable attention-based modelling, variational-autoencoder-based latent profiling, ensemble prediction, fairness evaluation and transfer validation within one occupational mental-health decision-support pipeline. Table 1 compares mental-health risk modelling studies with the proposed framework.

## 3. Methodology

This section presents the pipeline used to build an interpretable occupational mental-health risk index from heterogeneous workplace survey data. The workflow covers cohort construction, preprocessing, attention-based tabular learning, probability calibration, latent behavioural profiling, ensemble inference, fairness assessment, feature harmonization for external validation, transfer learning and reproducibility control. Early-career academics are used as a motivating high-pressure knowledge-work context; however, the empirical development and validation datasets are broader workplace mental-health and occupational burnout cohorts rather than direct early-career academic samples. The main data, preprocessing, evaluation and configuration details are given in Table 2, Table 3, Table 4 and Table 5.

### 3.1. Data Source, Cohort Construction and Outcome Definition

The analysis used a multi-country workplace mental health survey containing demographic, occupational, behavioural, access-to-care and help-seeking variables. The dataset was used as a development cohort for modelling occupational mental-health risk signals rather than as a direct sample of early-career academics. Table 2 summarizes the dataset, outcome, coverage and modelling role.

The supervised outcome is defined in Equation (1). It combines diagnosis, treatment, help-seeking and psychosocial support-need signals into one binary label. This outcome is interpreted as an operational indicator of elevated occupational mental-health support need rather than as a clinical diagnosis.

(1) $$y_{i}=I\left[\omega_{d}d_{i}+\omega_{t}t_{i}+\omega_{h}h_{i}+\omega_{s}q_{i}\geq \tau_{y}\right],\;q_{i}=\frac{1}{L_{i}}\sum_{\ell =1}^{L_{i}}\rho_{\ell }\;I\left[r_{i,\ell }\in R_{\ell }^{+}\right]$$

Here, $y_{i}$ is the outcome for respondent *i*, $d_{i}$ is reported diagnosis, $t_{i}$ is treatment or care use, $h_{i}$ is recent help-seeking and $q_{i}$ is the normalized psychosocial support-need score. The terms $r_{i,\ell }$, $R_{\ell }^{+}$, $\rho_{\ell }$, $\omega_{d},\omega_{t},\omega_{h},\omega_{s}$ and $\tau_{y}$ denote item response, elevated-risk response set, item weight, domain weights and threshold.

### 3.2. Preprocessing, Missingness Encoding and Mixed-Feature Representation

Table 3 summarizes preprocessing and training. Numerical variables were median-imputed and standardized using training statistics. Categorical variables were indexed and embedded. Missingness masks, composite features and filtered predictors were retained for modelling. These steps were applied consistently across model families to ensure that differences in performance reflected model behaviour rather than inconsistent feature preparation.

The mixed representation is defined in Equation (2). It concatenates categorical embeddings, standardized numerical features, missingness masks and composite indicators so that demographic, occupational, support-related and access-to-care signals can be represented in a unified modelling space.

(2) $$z_{i}=\left[\overset{C}{\underset{j=1}{⨁}}E_{j}o\left(c_{i,j}\right)\right]⊕\left[\overset{N}{\underset{k=1}{⨁}}\left(\frac{m_{i,k}n_{i,k}+\left(1-m_{i,k}\right)\mu_{k}^{\mathrm{tr}}-\mu_{k}^{\mathrm{tr}}}{\sigma_{k}^{\mathrm{tr}}+\epsilon },m_{i,k}\right)\right]⊕g_{i}$$

Here, $z_{i}$ is the respondent representation, $E_{j}$ is the embedding matrix, $o(c_{i,j})$ is the one-hot category vector, $n_{i,k}$ is the numerical value, $m_{i,k}$ is the missingness indicator, $\mu_{k}^{\mathrm{tr}}$ and $\sigma_{k}^{\mathrm{tr}}$ are training statistics and $g_{i}$ contains engineered composites. The composites used in Equation (2) are defined in Equation (3). They summarize workload, support, barriers and access-to-care conditions that are relevant across occupational mental-health contexts.

(3) $$g_{i}=\left[\eta_{w}\frac{\sum_{a\in W}\alpha_{a}{\tilde{x}}_{i,a}}{\sum_{a\in W}\left|\alpha_{a}\right|},\;\eta_{s}\frac{\sum_{b\in S}\beta_{b}{\tilde{x}}_{i,b}}{\sum_{b\in S}\left|\beta_{b}\right|},\;\eta_{b}\frac{\sum_{c\in B}\delta_{c}{\tilde{x}}_{i,c}}{\sum_{c\in B}\left|\delta_{c}\right|},\;\eta_{a}\frac{\sum_{d\in A}\kappa_{d}{\tilde{x}}_{i,d}}{\sum_{d\in A}\left|\kappa_{d}\right|}\right]$$

Here, W, S, B and A denote workload, support, barrier and access feature sets. The weights $\alpha_{a},\beta_{b},\delta_{c},\kappa_{d}$ control item contribution and $\eta_{w},\eta_{s},\eta_{b},\eta_{a}$ scale each domain.

### 3.3. Attention-Based Tabular Risk Model

**Figure 4:**
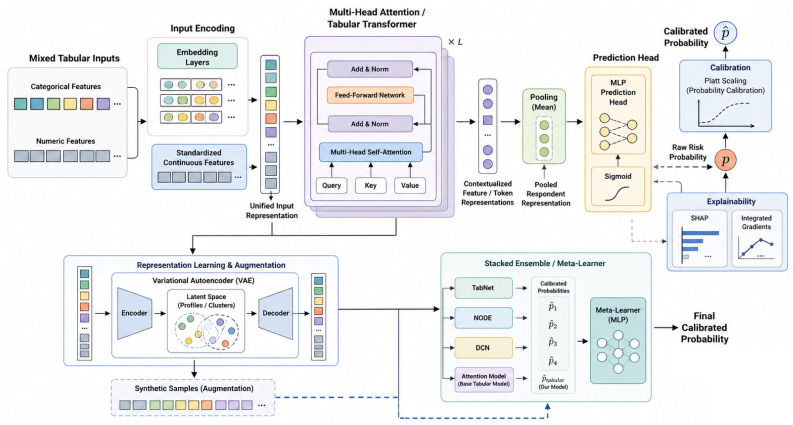
Mathematical overview of the proposed attention-based tabular learning framework. Arrows show data flow, colors distinguish the main framework modules, and ellipses indicate repeated tokens or components shown schematically for compactness.

The main model uses attention-based tabular learning to capture nonlinear and context-dependent feature relations among workplace, behavioural and support-related variables. Figure 4 summarizes the path from mixed inputs to attention learning, calibration, explanation, latent modelling and ensemble inference.

The contextual representation is defined in Equation (4). It uses multi-head attention, residual connections, layer normalization and feedforward transformation. This architecture allows the model to represent interactions among occupational stressors, support indicators, access-to-care variables and respondent characteristics.

(4) $$\begin{array}{c} A_{i,h}^{\left(\ell \right)}=\mathrm{softmax}\left(\frac{Q_{i,h}^{\left(\ell \right)}{\left(K_{i,h}^{\left(\ell \right)}\right)}^{⊤}+B_{h}^{\left(\ell \right)}}{\sqrt{d_{h}}}\right)V_{i,h}^{\left(\ell \right)},\\ U_{i}^{\left(\ell \right)}=\mathrm{LN}\left(H_{i}^{\left(\ell -1\right)}+W_{O}^{\left(\ell \right)}\left[A_{i,1}^{\left(\ell \right)}∥\cdots ∥A_{i,H}^{\left(\ell \right)}\right]\right),\\ H_{i}^{\left(\ell \right)}=\mathrm{LN}\left(U_{i}^{\left(\ell \right)}+W_{2}^{\left(\ell \right)}\;\varphi \left(W_{1}^{\left(\ell \right)}U_{i}^{\left(\ell \right)}+b_{1}^{\left(\ell \right)}\right)+b_{2}^{\left(\ell \right)}\right) \end{array}$$

Here, $H_{i}^{\left(\ell \right)}$ is the layer-*ℓ* representation, initialized from $z_{i}$. The terms Q, K and V are attention projections; $d_{h}$ is head dimension; B, W and b are trainable parameters; and LN denotes layer normalization. The pooled respondent vector and raw probability are defined in Equation (5).

(5) $$\begin{array}{c} \alpha_{i}=\mathrm{softmax}\left(v_{p}^{⊤}\tanh\left(W_{p}H_{i}^{\left(L\right)}+b_{p}\right)\right),\\ {\bar{h}}_{i}=\sum_{t=1}^{T}\alpha_{i,t}H_{i,t}^{\left(L\right)},\\ {\hat{p}}_{i}=\sigma \left(w_{r}^{⊤}\mathrm{Dropout}\left({\bar{h}}_{i}\right)+b_{r}\right) \end{array}$$

Here, $\alpha_{i}$ is the pooling-weight vector, ${\bar{h}}_{i}$ is the respondent embedding, *T* is the number of tokens and ${\hat{p}}_{i}$ is the uncalibrated risk probability. The supervised objective is shown in Equation (6). It combines weighted focal loss, label smoothing and regularization to support learning under class imbalance while reducing overconfident predictions.

(6) $$L_{\sup}=-\frac{1}{M}\sum_{i=1}^{M}\left[\lambda_{1}y_{i}^{\epsilon }{\left(1-{\hat{p}}_{i}\right)}^{\gamma }\log\left({\hat{p}}_{i}+\epsilon \right)+\lambda_{0}\left(1-y_{i}^{\epsilon }\right){\hat{p}}_{i}^{\gamma }\log\left(1-{\hat{p}}_{i}+\epsilon \right)\right]+\Omega \left(\Theta \right)$$

Here, *M* is batch size, $y_{i}^{\epsilon }$ is the smoothed label, $\lambda_{1}$ and $\lambda_{0}$ are class weights, γ is the focal parameter, ${\hat{p}}_{i}$ is from Equation (5) and Ω(Θ) is regularization.

### 3.4. Probability Calibration and Risk Index Mapping

The raw probability from Equation (5) is calibrated before risk-index conversion. Calibration aligns predicted probabilities with observed event frequencies and is therefore essential before using model outputs for risk communication or decision support. The calibrated probability and risk index are defined in Equation (7).

(7) $$\begin{array}{cc} s_{i} & =\;\sigma \left[a\;\mathrm{logit}\left(\mathrm{clip}\left({\hat{p}}_{i},\epsilon ,1-\epsilon \right)\right)+b\right],\\ {\mathrm{RI}}_{i} & =\;100\cdot \frac{s_{i}-s_{\min}^{\mathrm{val}}}{s_{\max}^{\mathrm{val}}-s_{\min}^{\mathrm{val}}+\epsilon },\\ {\mathrm{Band}}_{i} & =\;\sum_{q=1}^{4}q\;I\left[\kappa_{q-1}\leq {\mathrm{RI}}_{i}<\kappa_{q}\right] \end{array}$$

Here, $s_{i}$ is the calibrated probability, ${\hat{p}}_{i}$ is from Equation (5), *a* and *b* are validation-fitted calibration parameters, ${\mathrm{RI}}_{i}$ is the 0–100 score and ${\mathrm{Band}}_{i}$ is the risk band. Operationally, calibration was performed on the validation partition after model training. The uncalibrated neural-network output was first clipped to avoid numerical instability, transformed using the logit function and then passed through a validation-fitted Platt-scaling model. The calibrated probability was then linearly mapped to a 0–100 risk-index scale using the minimum and maximum calibrated probabilities observed in the validation set. This procedure separates three quantities: the raw model score, the calibrated probability and the final communication-oriented risk index. The score is intended as an interpretable occupational risk index for support prioritization rather than as a diagnostic measure or automatic intervention rule.

### 3.5. Latent Behavioural Profiling and Synthetic Data Generation

A variational autoencoder is used to learn latent occupational–behavioural profiles linked to support, workload interference, stigma and access-to-care patterns. This latent representation strategy is consistent with broader deep-learning evidence showing that sequential recurrent learning can extract compact patterns from complex input spaces [54]. Hybrid CNN-DNN mechanisms provide related evidence that complementary neural components can improve discriminative representation learning [55]. Topology-aware graph convolution further supports the modelling of structured dependencies across complex feature spaces [22]. Temporal network modelling also shows how evolving relationships can be represented for predictive learning under dynamic conditions [23]. Unsupervised anomaly detection provides additional support for discovering compact high-risk patterns without relying only on predefined labels [25]. These profiles are intended to summarize recurring workplace risk configurations rather than to define fixed clinical subtypes. Latent subgroups were interpreted descriptively by comparing cluster-level distributions of calibrated risk, work interference, stress burden, support availability, care-access variables and stigma-related indicators. Therefore, a cluster label does not represent a diagnosis or a stable psychological category. Instead, it represents a recurring pattern of workplace survey responses that may help explain whether elevated risk is mainly associated with work interference, limited support, care-access barriers or related occupational conditions. The mixed-type VAE objective is given in Equation (8).

(8) $$\begin{array}{ccc} L_{\mathrm{VAE}} & = & E_{q_{\phi }\left(u_{i}|z_{i}\right)}\left[\sum_{k\in N}\frac{{\left({\tilde{n}}_{i,k}-{\hat{n}}_{i,k}\right)}^{2}}{2\sigma_{k}^{2}}+\sum_{j\in C}\mathrm{CE}\left(c_{i,j},{\hat{\pi }}_{i,j}\right)\right]\\ & & +\beta \;\mathrm{KL}\left[q_{\phi }\left(u_{i}|z_{i}\right)\;∥\;p\left(u_{i}\right)\right],\\ q_{\phi }\left(u_{i}|z_{i}\right) & = & N\left(\mu_{\phi }\left(z_{i}\right),\mathrm{diag}\left[\sigma_{\phi }^{2}\left(z_{i}\right)\right]\right) \end{array}$$

Here, $L_{\mathrm{VAE}}$ combines numerical reconstruction, categorical reconstruction and KL regularization. The latent vector is $u_{i}$ and β controls the reconstruction–regularization balance. Latent profile assignment is defined in Equation (9).

(9) $$\begin{array}{cc} p\left(u_{i}\right) & =\;\sum_{k=1}^{K}\pi_{k}\;N\left(u_{i}|\mu_{k},\Sigma_{k}\right),\\ \gamma_{i,k} & =\;\frac{\pi_{k}\;N\left(u_{i}|\mu_{k},\Sigma_{k}\right)}{\sum_{r=1}^{K}\pi_{r}\;N\left(u_{i}|\mu_{r},\Sigma_{r}\right)},\\ C_{i} & =\;\arg\max_{k}\gamma_{i,k} \end{array}$$

Here, *K* is the number of clusters, $\pi_{k}$ is the mixture weight, $\mu_{k}$ and $\Sigma_{k}$ are cluster parameters, $\gamma_{i,k}$ is cluster responsibility and $C_{i}$ is the assigned profile. Synthetic samples are generated and checked using Equation (10). They are used only for robustness and augmentation experiments after validation and not as a substitute for real workplace mental-health observations.

(10) $$u_{k}^{\left(s\right)}∼N\left(\mu_{k},\Sigma_{k}\right),\;{\tilde{x}}^{\left(s\right)}∼p_{\psi }\left(x|u_{k}^{\left(s\right)}\right),\;\Delta_{\mathrm{dist}}=\sum_{m=1}^{P}\xi_{m}D_{m}\left(P_{\mathrm{real}}^{m},P_{\mathrm{syn}}^{m}\right)$$

Here, $u_{k}^{\left(s\right)}$ is a sampled latent vector, ${\tilde{x}}^{\left(s\right)}$ is the decoded record and $\Delta_{\mathrm{dist}}$ measures real–synthetic distributional mismatch.

### 3.6. Stacked Ensemble, Transfer Learning and Validation Metrics

The final model combines attention-based tabular learning, TabNet-style feature selection, neural oblivious decision ensembles and deep-cross interaction models. This design follows a stacked ensemble rationale where complementary learners are combined to improve classification robustness across complex structured inputs [56]. It also follows a multihead feature-extraction rationale where different representation heads capture complementary aspects of heterogeneous survey data [57]. This ensemble design is used to compare complementary representations of structured workplace survey data while improving robustness beyond a single neural architecture. Table 4 lists the evaluation, calibration, latent profiling, fairness and transfer procedures.

The stacked ensemble is defined in Equation (11). Bootstrap confidence intervals and the DeLong test were selected because they address complementary aspects of model evaluation. Bootstrap confidence intervals provide a non-parametric estimate of uncertainty for performance metrics such as AUC, average precision, F1 score, Brier score and calibration error by repeatedly resampling the test set with replacements. This strategy supports robust performance reporting when metric distributions may be non-normal or influenced by class imbalance. Recent deep-learning work on complex biomedical signal classification has similarly emphasized rigorous performance evaluation for reliable model validation, including entropy-informed residual-network evaluation for fine-grained EEG driver-state recognition [50]. In contrast, the DeLong test was used specifically for paired ROC-AUC comparison because it accounts for the correlation between models evaluated on the same test respondents. Together, bootstrap confidence intervals quantify the stability of individual performance estimates, whereas the DeLong test evaluates whether observed AUC differences between competing models are statistically meaningful.

(11) $$\begin{array}{cc} m_{i} & =\;{\left[{\hat{p}}_{i}^{\mathrm{Tab}},{\hat{p}}_{i}^{\mathrm{TabNet}},{\hat{p}}_{i}^{\mathrm{NODE}},{\hat{p}}_{i}^{\mathrm{DCN}},s_{i}\right]}^{⊤},\\ {\hat{p}}_{i}^{\mathrm{meta}} & =\;\sigma \left(w_{m}^{⊤}m_{i}+b_{m}\right),\\ L_{\mathrm{meta}} & =\;-\frac{1}{M}\sum_{i=1}^{M}\left[y_{i}\log{\hat{p}}_{i}^{\mathrm{meta}}+\left(1-y_{i}\right)\log\left(1-{\hat{p}}_{i}^{\mathrm{meta}}\right)\right]+\lambda_{m}{∥w_{m}∥}_{2}^{2} \end{array}$$

**Figure 5:**
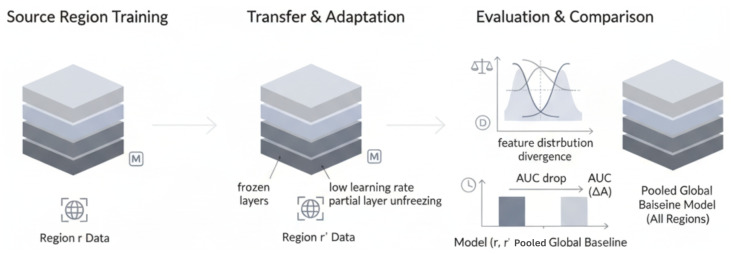
Cross-domain transfer learning and domain generalization process, including source-domain training, partial layer freezing, target-domain fine-tuning, target evaluation, divergence measurement, AUC-drop calculation and comparison against pooled global and target-only baselines.

Here, $m_{i}$ contains component predictions and $s_{i}$ from Equation (7). The meta-learner produces ${\hat{p}}_{i}^{\mathrm{meta}}$ using regularized logistic fusion. The transfer-learning process is shown in Figure 5.

The adaptation objective is given in Equation (12).

(12) $$\begin{array}{cc} \Theta_{s}^{*} & =\;\arg\min_{\Theta }L_{s}\left(\Theta ;D_{s}\right),\\ \Theta_{s\to t}^{*} & =\;\arg\min_{\Theta }\left[L_{t}\left(\Theta ;D_{t}\right)+\lambda_{\theta }{∥\Theta_{F}-\Theta_{s,F}^{*}∥}_{2}^{2}+\lambda_{d}\;{\mathrm{MMD}}^{2}\left(H_{s},H_{t}\right)\right],\\ \Delta A_{s\to t} & =\;A\left(\Theta_{s}^{*},D_{t}^{\mathrm{test}}\right)-A\left(\Theta_{s\to t}^{*},D_{t}^{\mathrm{test}}\right) \end{array}$$

Here, $\Theta_{s}^{*}$ is the source model, $\Theta_{s\to t}^{*}$ is the adapted model, $D_{s}$ and $D_{t}$ are source and target datasets and MMD measures domain alignment. Fairness and subgroup reliability are assessed using Equation (13).

(13) $$\begin{array}{cc} \Delta_{\mathrm{AUC}} & =\;\max_{g\in G}A_{g}-\min_{g\in G}A_{g},\\ \Delta_{\mathrm{TPR}} & =\;\max_{g\in G}\frac{\sum_{i:g_{i}=g}I\left[{\hat{y}}_{i}=1,y_{i}=1\right]}{\sum_{i:g_{i}=g}I\left[y_{i}=1\right]+\epsilon }-\min_{g\in G}\frac{\sum_{i:g_{i}=g}I\left[{\hat{y}}_{i}=1,y_{i}=1\right]}{\sum_{i:g_{i}=g}I\left[y_{i}=1\right]+\epsilon },\\ \Delta_{\mathrm{CAL}} & =\;\max_{g\in G}\left|E\left[s_{i}-y_{i}|g_{i}=g\right]\right| \end{array}$$

Here, G is the subgroup set, $A_{g}$ is subgroup AUC, ${\hat{y}}_{i}$ is the predicted label, $y_{i}$ is from Equation (1) and $s_{i}$ is from Equation (7).

### 3.7. Interpretability, Robustness and Reproducibility

Interpretability used global and local attribution, attention inspection, integrated gradients and perturbation tests. These analyses were included to evaluate whether the model relied on plausible occupational and support-related signals rather than unstable artefacts. Table 5 lists the main settings and reproducibility controls.

The attribution framework is defined in Equation (14).

(14) $$\begin{array}{cc} f\left(x_{i}\right) & =\;\phi_{0}+\sum_{j=1}^{D}\phi_{i,j},\\ {\mathrm{IG}}_{i,j} & =\;\left(x_{i,j}-x_{j}^{\prime }\right)\int_{0}^{1}\frac{\partial f\left(x^{\prime }+\alpha \left(x_{i}-x^{\prime }\right)\right)}{\partial x_{i,j}}d\alpha ,\\ S_{\phi } & =\;\frac{2}{K_{\mathrm{cv}}\left(K_{\mathrm{cv}}-1\right)}\sum_{a<b}{\mathrm{corr}}_{\mathrm{rank}}\left(\left|\phi^{\left(a\right)}\right|,\left|\phi^{\left(b\right)}\right|\right) \end{array}$$

Here, $\phi_{i,j}$ is the SHAP contribution, ${\mathrm{IG}}_{i,j}$ is integrated-gradient attribution and $S_{\phi }$ measures attribution-rank stability across folds. Perturbation robustness is defined in Equation (15).

(15) $$\Delta A_{j}=A\left(X,y\right)-A\left(X_{\setminus j}^{\pi },y\right),\;R_{j}=\frac{\Delta A_{j}}{\sum_{r=1}^{D}\left|\Delta A_{r}\right|+\epsilon }$$

Here, $\Delta A_{j}$ is the AUC change after perturbing feature *j* and $R_{j}$ is normalized perturbation importance. The final workflow links Table 2, Table 3, Table 4 and Table 5 and Equations (1)–(15) into one computational system for calibrated, interpretable and subgroup-aware occupational risk modelling.

### 3.8. Comparison with State-of-the-Art Baseline Models

The proposed calibrated attention-latent ensemble was compared with conventional and neural baselines for heterogeneous tabular occupational-risk prediction. All models used the same data partitions and preprocessing steps and metrics defined in Section 3.2 and Table 4. Support vector machine classification was included because margin-based methods remain useful structured-data baselines and active-learning extensions can improve decision-boundary optimization [42]. The protocol also considered uncertainty-aware sampling for reliability assessment [60]. Robust KNN-based classification was included as an additional conventional comparator [61]. Adaptive optimization and false-alarm-aware classification further motivated the robustness checks used in the evaluation design [62]. Threshold reliability was also assessed because false alarms can distort decision-support outputs in risk modelling [63]. Hyperparameters were tuned on validation data and final estimates were computed once on the test set. Bootstrap confidence intervals quantified metric uncertainty and the DeLong test supported paired ROC-AUC comparison. Table 6 summarizes the protocol.

Performance gain over each baseline is defined in Equation (16).

(16) $$\Delta M_{b}=M_{\mathrm{prop}}-M_{b},\;G_{b}=\frac{M_{\mathrm{prop}}-M_{b}}{|M_{b}|+\epsilon }\times 100,\;\Delta_{\mathrm{fair},b}=\Gamma_{\mathrm{prop}}-\Gamma_{b}$$

Here, $M_{\mathrm{prop}}$ and $M_{b}$ are proposed-model and baseline scores, $\Delta M_{b}$ is the absolute difference, $G_{b}$ is percentage gain and $\Delta_{\mathrm{fair},b}$ is the fairness-gap difference. For Brier score and ECE, lower values indicate better performance.

### 3.9. Cross-Dataset Evaluation Protocol

External generalization was tested using the HackerEarth Employee Burnout Challenge dataset as an independent occupational burnout cohort with the OSMI survey serving as the development dataset. The external cohort includes train.csv with 22,750 records and 9 variables and test.csv with 12,250 records and 8 variables, covering employee identifier, joining date, gender, company type, work-from-home setup, designation, resource allocation, mental fatigue score and Burn Rate. Because the OSMI and HackerEarth datasets used different survey instruments, external evaluation used conceptual feature harmonization rather than direct item-level matching. Variables were aligned only when they represented comparable occupational-health domains, including demographics, employment context, work arrangement, workload intensity, fatigue or stress burden and support-related proxies. Features without a defensible conceptual counterpart were excluded from the main harmonized model and retained only for sensitivity analysis. The continuous HackerEarth Burn Rate was converted into a binary high-risk label using the upper tertile of the training distribution with a 0.50 threshold used as a sensitivity check.

Table 7 summarizes source-only transfer, target-only baseline, pooled learning, target-adapted fine-tuning, feature harmonization, label conversion, calibration transfer and fairness checks. All settings followed Section 3.2, Section 3.4 and Section 3.6. The transfer-learning procedure had four stages: source-model training on OSMI, direct evaluation on the harmonized HackerEarth representation to quantify source-only transfer under dataset shift, target adaptation using labelled HackerEarth training data with regularization toward the source solution and target recalibration plus test evaluation using discrimination, calibration and fairness metrics. This design evaluates whether broad occupational risk signals can transfer after harmonization and recalibration without claiming that OSMI mental-health items and HackerEarth burnout variables are identical measurements or that source-only results are deployment-ready evidence.

The external burnout label is defined in Equation (17).

(17) $$y_{i}^{\mathrm{HE}}=I\left[b_{i}\geq Q_{0.67}\left(B_{\mathrm{train}}\right)\right]$$

Here, $y_{i}^{\mathrm{HE}}$ is the high-burnout label, $b_{i}$ is Burn Rate and $Q_{0.67}\left(B_{\mathrm{train}}\right)$ is the training-set 67th percentile. The cross-dataset adaptation objective is defined in Equation (18).

(18) $$\Theta_{\mathrm{OSMI}\to \mathrm{HE}}^{*}=\arg\min_{\Theta }\left[L_{\mathrm{OSMI}}\left(\Theta ;D_{\mathrm{OSMI}}\right)+\lambda_{\mathrm{HE}}L_{\mathrm{HE}}\left(\Theta ;D_{\mathrm{HE}}^{\mathrm{train}}\right)+\lambda_{m}{\mathrm{MMD}}^{2}\left(H_{\mathrm{OSMI}},H_{\mathrm{HE}}\right)+\lambda_{c}\left|{\mathrm{ECE}}_{\mathrm{OSMI}}-{\mathrm{ECE}}_{\mathrm{HE}}\right|\right]$$

Here, $\Theta_{\mathrm{OSMI}\to \mathrm{HE}}^{*}$ is the adapted model, $L_{\mathrm{OSMI}}$ and $L_{\mathrm{HE}}$ are source and target losses, MMD aligns hidden representations and the ECE term controls calibration consistency. External generalization is summarized by Equation (19).

(19) $$\Delta_{\mathrm{CD}}=\left[A_{\mathrm{within}}-A_{\mathrm{cross}}\right]+\omega_{e}\left[{\mathrm{ECE}}_{\mathrm{cross}}-{\mathrm{ECE}}_{\mathrm{within}}\right]+\omega_{g}\left[\Gamma_{\mathrm{cross}}-\Gamma_{\mathrm{within}}\right]$$

Here, $\Delta_{\mathrm{CD}}$ is the degradation score, *A* is AUC, ECE is calibration error, Γ is the fairness gap and $\omega_{e},\omega_{g}$ weight calibration and fairness degradation.

### 3.10. Hardware and Software Environment

To improve reproducibility, all experiments were implemented in a controlled Python 3.10.12 environment. environment using fixed random seeds, stratified train–validation–test splits and deterministic backend settings where available. The same hardware and software stack was used for the proposed model and all baselines, including preprocessing, attention-based tabular modelling, VAE latent profiling, ensemble learning, calibration, interpretability analysis and transfer evaluation. The computational environment is summarized in Table 8 and package versions, configuration files and experiment logs were retained for replication.

## 4. Results

This section reports the empirical findings produced by the proposed methodological pipeline. The results are presented in the same workflow order as the methodology, covering calibration, discrimination, baseline comparison, interpretability, latent profiling, fairness assessment, cross-region transfer and external cross-dataset evaluation. This organization ensures that the reported evidence follows a coherent sequence from model development and validation to interpretability, subgroup analysis, transfer evaluation and external dataset assessment. Throughout this section, the findings are interpreted as evidence for occupational mental-health risk modelling from heterogeneous workplace survey data with relevance to knowledge-work settings rather than as direct empirical measurement of early-career academic populations.

### 4.1. Calibration, Threshold Performance and Risk-Index Mapping

Table 9 evaluates whether the calibrated probabilities produced by Equation (7) correspond to observed outcome frequencies and can therefore support an interpretable risk-index scale. The calibration bins show close alignment between mean predicted probability and observed fraction positive across the full probability range. In the lowest bin, the mean predicted probability is 0.069575, while the observed fraction positive is 0.076923 across 78 respondents. This indicate that the model does not substantially inflate low-risk estimates, in the intermediate range the 0.20–0.50 bin have a mean prediction of 0.358531 and also an observed fraction positive of 0.422222 across 45 respondents, showing modest underestimation among ambiguous cases. The 0.50–0.80 bin shows stronger agreement with predicted probability 0.688924 and observed fraction positive 0.693877 across 49 respondents. The highest-risk bin remains practically well-calibrated with mean prediction 0.886547 and observed fraction positive 0.862500 across 80 respondents.

Thesame table also reports strong discrimination with AUC ROC = 0.88199 and average precision = 0.86884. These values shows that calibration did not remove the ranking strength needed to identify respondents with elevated occupational mental-health support needs. The calibrated probabilities are then mapped into a 0–100 risk index and four operational risk bands. Very low risk corresponds to scores 1.6–11.2, low risk to 11.2–55.4, moderate risk to 55.4–85.8 and high risk to 85.8–96.3. This mapping converts probabilistic model outputs into an operational scale that can support monitoring, outreach prioritization and further assessment, while remaining distinct from a clinical diagnosis.

### 4.2. Predictive Discrimination and Attention-Based Interpretability

**Figure 6:**
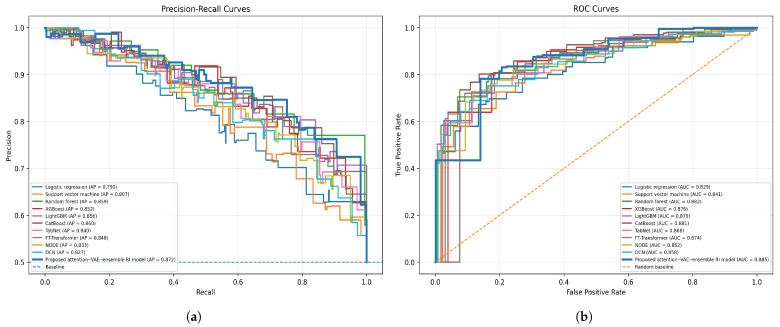
Precision–recall and ROC curves summarising predictive performance across evaluated model families, with overlapping curve segments indicating comparable model behaviour. (**a**) Precision–recall curve for evaluated baseline and proposed occupational risk models. (**b**) ROC curve for evaluated baseline and proposed occupational risk models.

Figure 6 summarizes discrimination across the evaluated baseline and proposed models using the precision–recall and ROC protocols defined in Table 4. The precision–recall curves show that several models retrieve positive cases effectively with the strongest models reaching average precision values near 0.87. This result is important because the positive class represents respondents with elevated likelihood of occupational mental-health support need. Precision–recall performance therefore provides a more relevant view of elevated-risk retrieval than accuracy alone. The ROC curves show similarly strong separation between positive and negative cases with leading models reaching AUC values near 0.88. The curves are relatively clustered, indicating that multiple tabular learning methods are competitive under the same preprocessing, splitting and validation protocol.

**Figure 7:**
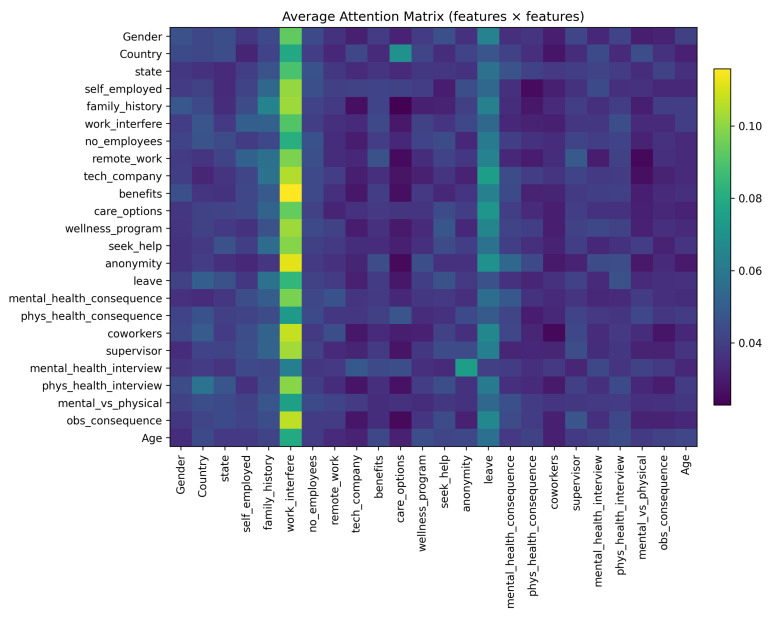
Average attention matrix showing feature-dependency structure learned by the tabular model.

The proposed attention-based risk model remains among the strongest performers, supporting the representation and prediction logic in Equations (4) and (5). Taken together, the two panels suggests that the model still preserves ranking quality while keeping strong retrieval of likely high-risk respondents. This supports using calibrated predictions as the basis for downstream risk indexing, latent-profile analysis, perturbation testing and fairness evaluation. Figure 7 provides an interpretability check for the attention module. The highest attention concentration appears around work_interfere, indicating that the model learns a focused dependency structure instead of spreading importance evenly across every input. This result agrees with the later perturbation findings, where work interference produces the largest performance drop when it is disrupted.

### 4.3. State-of-the-Art Baseline Comparison

Table 10 provides the main comparative evaluation because it directly tests the proposed attention-VAE-ensemble RI model against linear, kernel, tree-based, boosting and deep tabular baselines. The comparison follows the baseline protocol in Section 3.8 and uses the gain logics in Equation (16). The proposed model achieves the best overall discrimination with AUC = 0.885, AP = 0.872 and F1 = 0.808. Random forest is the closest conventional comparator with AUC = 0.882, AP = 0.859 and F1 = 0.802. CatBoost is also competitive with AUC = 0.881, AP = 0.860 and F1 = 0.801. The proposed model therefore gives modest AUC gains but clearer improvement in average precision and F1.

The calibration and fairness columns are especially important because the proposed framework is intended for responsible risk scoring rather than discrimination alone. The proposed model achieves the lowest Brier score, 0.145, and the lowest ECE, 0.022. CatBoost is the closest calibrated baseline with Brier = 0.148 and ECE = 0.030, while random forest has Brier = 0.153 and ECE = 0.039. The proposed model also gives the smallest fairness gap, 0.169, compared with 0.184 for CatBoost, 0.181 for FT–Transformer and 0.206 for random forest. These results indicate that the proposed framework improves probability reliability and subgroup consistency in addition to maintaining competitive discrimination.

### 4.4. Interaction Structure, Perturbation Sensitivity and Cross-Validation Stability

**Figure 8:**
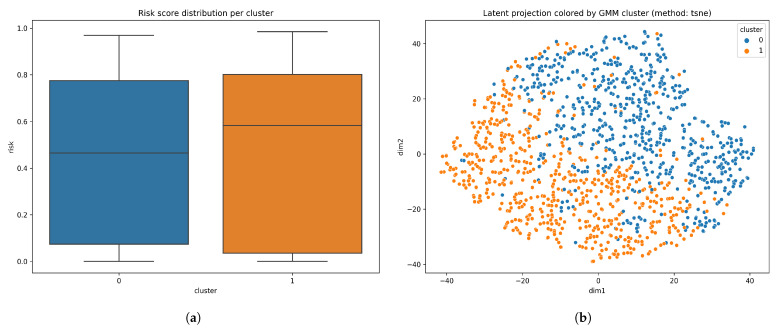
Cluster-level risk distributions and latent-space separation across inferred behavioural profiles. (**a**) Risk-score distribution across latent behavioural clusters identified by the GMM model. (**b**) Latent-space projection showing partial separation and boundary mixing across clusters.

**Figure 9:**
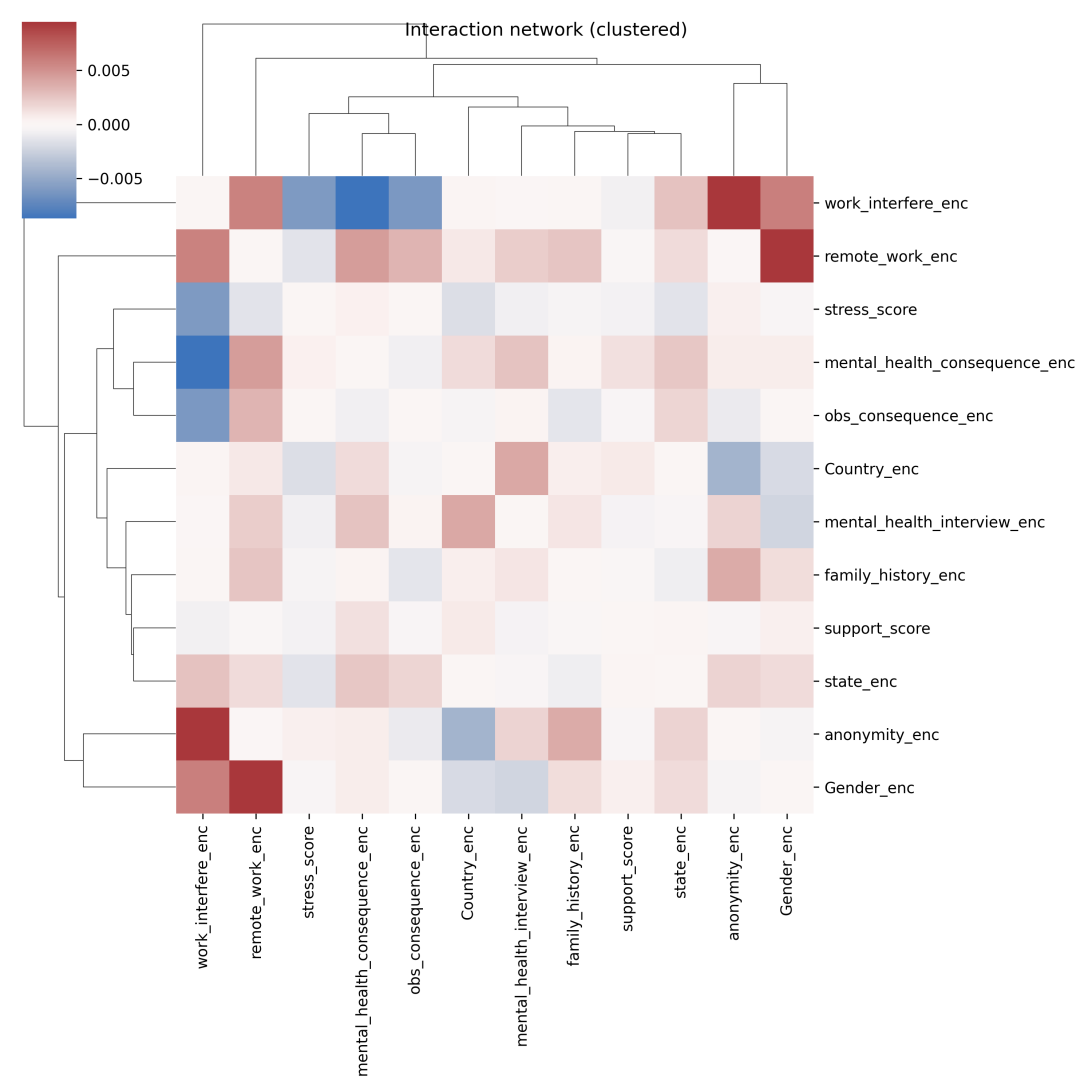
Clustered interaction network heatmap showing reinforcing and compensatory feature relationships.

Figure 8 and Figure 9, and Table 11 evaluate whether the proposed model relies on stable and interpretable feature relationships. The interaction heatmap shows clustered dependencies among occupational, demographic and support-related variables, indicating that the model does not distribute importance uniformly across all inputs. Instead, several feature groups form localized interaction patterns, supporting attention-guided hidden-pattern discovery in heterogeneous behavioural data. This interpretation is conceptually consistent with attention-enhanced abnormal-pattern identification methods in other complex data domains, where deep representation learning and attention mechanisms have been used to detect subtle hidden signals and improve robustness under difficult distributional conditions [16].

The strongest listed interaction effects involve work_int with anon and gender with remote, both with interaction values of 0.009. The gender-remote interaction is statistically significant with p=0.020 and the country-mh_intervw interaction is also significant with p=0.020. These results suggest that risk prediction is influenced not only by individual features but also by contextual relationships between work arrangement, demographic background and help-seeking attitudes.

Perturbation analysis further confirms the importance of work interference. When work_int is perturbed, AUC decreases from 0.844 to 0.657, corresponding to a 22.1% relative reduction. In contrast, perturbing stress, support, country and anon produces only minor changes in AUC, suggesting that these variables are either more redundant with other predictors or less individually decisive under the perturbation protocol.

The cross-validation results in Table 11 should be interpreted as a robustness analysis rather than as the main held-out benchmark comparison. The fixed held-out baseline-comparison experiment reports the final proposed-model performance under one predefined test partition, whereas the five-fold cross-validation results summarize performance variability across multiple train–test partitions. Therefore, the fixed held-out AUC of 0.885 and the five-fold mean test AUC of 0.809±0.044 are not contradictory; they represent different validation settings. The lower cross-validation mean provides a more conservative estimate of generalization and indicates moderate sensitivity to data partitioning. Across folds, validation AUC remains 0.846±0.019, validation AP remains 0.794±0.035, test AUC is 0.809±0.044 and test F1 is 0.746±0.057.

### 4.5. Latent Behavioural Profiles and Synthetic Validity

Table 12 and Table 13 summarize the latent profile results from the VAE-GMM procedure in Equations (8) and (9). Two occupational–behavioural profiles are identified. Cluster $C_{0}$ is characterized by rare work interference, smaller organizations, positive coworker support, positive supervisor support and lower average risk. Cluster $C_{1}$ shows more frequent work interference, larger organizations, partial coworker support, weaker supervisor support and higher average risk. The cluster summary confirms this difference with Cluster 0 containing 649 respondents and average risk 0.448, while Cluster 1 contains 610 respondents and average risk 0.487. The visual panels support this interpretation by showing higher risk spread in Cluster 1 and partial latent-space separation with boundary mixing.

Synthetic validity is mixed and should be interpreted conservatively. Random forest baseline performance is AUC = 0.882 and AP = 0.859, while the augmented version gives AUC = 0.879 and AP = 0.864. This suggests that synthetic augmentation slightly improves ranking precision but does not improve overall discrimination. Continuous-variable fidelity is weaker, especially for age with KS = 0.778 and EMD $=1.2\times 10^{8}$ and stress_score with KS = 0.589 and EMD = 0.530. Categorical variables show more reasonable agreement, indicating that synthetic records are more defensible for robustness checks than for unrestricted replacement of real workplace observations.

### 4.6. Feature Ablation, Subgroup Fairness and Regional Transfer

**Figure 10:**
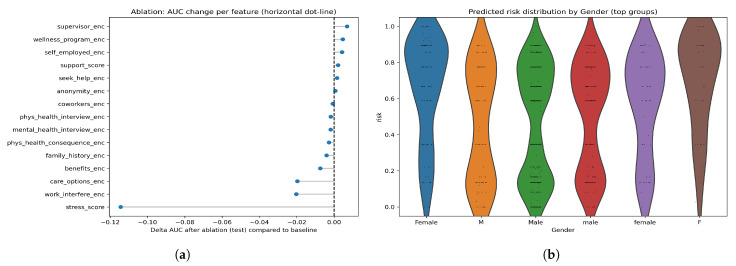
Ablation effects and fairness-related variation in predicted occupational mental-health risk. (**a**) Ablation analysis showing AUC changes after feature removal or perturbation. (**b**) Predicted risk distributions showing subgroup variation across gender categories, where the dots represent individual sample-level predicted risk values.

Figure 10, and Table 14 and Table 15 evaluate whether the models are driven by plausible occupational signals and whether reliability varies across subgroups and regions. The ablation results show that stress_score is the dominant individual predictor because its removal reduces AUC by 0.114. The next strongest effects are work_interfere_enc and care_options_enc, each reducing AUC by 0.020, while benefits_enc has a smaller effect of −0.007. These values agree with the attention and perturbation findings, confirming that stress burden, work interference and care access are central model drivers.

The subgroup results show uneven country-level reliability. Germany has the highest listed subgroup AUC at 0.903, followed by the United Kingdom at 0.880 and the United States at 0.868. Canada is lower at 0.734, indicating a meaningful subgroup performance gap that should not be hidden by aggregate metrics. The regional transfer table further supports this conclusion. The United States has self-transfer AUC = 0.698 and mean cross-region AUC = 0.637, while Germany has self-transfer AUC = 0.677 and mean cross-region AUC = 0.579. The United Kingdom performs weakly in self-transfer with AUC = 0.465 and only modestly as a source with mean cross-region AUC = 0.492. These findings support local recalibration, subgroup reporting and source-to-target adaptation as defined in Equation (12).

### 4.7. Cross-Dataset Evaluation on the HackerEarth Burnout Dataset

Table 16 reports the external cross-dataset evaluation using the independent HackerEarth Employee Burnout Challenge dataset. The experiment follows the harmonization and adaptation protocol in Section 3.9. Source-only transfer performs weakly with AUC = 0.642, AP = 0.617, ECE = 0.128 and fairness gap = 0.214. This confirms substantial dataset shift and shows that direct deployment of the OSMI-trained source model would be unreliable without target-domain adaptation. Source calibration alone does not improve ranking and slightly worsens ECE to 0.132, while target recalibration reduces ECE to 0.052 but leaves AUC and AP unchanged at 0.642 and 0.617.

Target-aware models perform substantially better. The target-only baseline reaches AUC = 0.932, AP = 0.925, ECE = 0.041 and fairness gap = 0.092. Pooled learning remains strong but slightly weaker with AUC = 0.919 and AP = 0.913, suggesting that source-domain data add coverage but may dilute target-specific signals. Target-adapted fine-tuning improves performance to AUC = 0.935, AP = 0.929, ECE = 0.038 and fairness gap = 0.086. The final harmonized and adapted OSMI-HackerEarth model performs best overall, achieving AUC = 0.941, AP = 0.936, ECE = 0.029 and fairness gap = 0.078. These results support the cross-dataset objective in Equation (18), showing that external performance is strongest when conceptual harmonization, target labels, calibration control and adaptation are combined. The findings should therefore be interpreted as evidence for transferable occupational risk modelling after target-domain adaptation, not as evidence that an OSMI-only model generalizes directly to all occupational burnout settings.

## 5. Discussion and Comparison

Table 17 compares the proposed framework with recent studies that address occupational stress, burnout, sickness absence associated with mental disorders and explainable workplace mental-health prediction. The comparison indicates that contemporary research increasingly integrates survey-based workplace data with machine-learning models, explainability techniques and empirical validation procedures. In relation to these studies, the proposed framework provides a broader occupational mental-health modelling system by jointly incorporating calibrated risk-index mapping, attention-based tabular learning, VAE-GMM latent profiling, subgroup fairness assessment and cross-dataset transfer validation. This comparison positions the study as a general occupational risk-modelling contribution with relevance to high-pressure knowledge-work settings, including but not limited to early-career academic contexts.

### 5.1. Predictive Performance, Calibration and Risk-Index Utility

The proposed model demonstrates competitive predictive performance against recent occupational and workplace mental-health prediction studies while also adding calibrated risk-index utility for decision support. In Table 10, the proposed attention-VAE-ensemble RI model achieves AUC = 0.885, AP = 0.872 and F1 = 0.808, which places it above random forest, CatBoost, LightGBM, XGBoost and deep tabular baselines under the same evaluation protocol. The AUC gain over random forest is small because random forest already reaches AUC = 0.882, yet the proposed model improves AP from 0.859 to 0.872 and F1 from 0.802 to 0.808, which is important because AP better reflects retrieval of respondents with elevated support needs.

Recent studies report similar aggregate discrimination, although they usually emphasize classification performance more than calibrated decision support. Hasan et al. reported ensemble accuracy of 90.32% for occupational stress detection, which confirms the usefulness of ensemble learning for workplace stress prediction [71]. Jeong et al. reported random forest ROC-AUC = 0.904 for burnout-syndrome risk prediction using KOSS variables, which further shows that structured occupational indicators can produce strong discrimination [72]. Mokheleli et al. reported XGBoost accuracy = 91% and ROC-AUC = 90% using OSMI workplace mental-health data, which provides another relevant benchmark for workplace mental-health prediction [34].

The main distinction of the present study is that Table 9 converts predicted probabilities into calibrated risk bands with AUC ROC = 0.88199, AP = 0.86884, Brier score = 0.145 and ECE = 0.022. This calibration layer makes the model more suitable for institutional decision support because probability reliability is needed before assigning low, moderate or high outreach categories. The resulting risk index should therefore be interpreted as an operational support-prioritization tool rather than as a diagnostic instrument.

### 5.2. Interpretability, Latent Profiles and Occupational Risk Mechanisms

The interpretability results indicate that the proposed framework identifies plausible occupational mechanisms rather than only maximizing aggregate accuracy. Figure 7 shows that attention is concentrated around work_interfere, while Table 11 confirms that perturbing work_int reduces AUC from 0.844 to 0.657, which corresponds to a 22.1% performance loss. Table 14 independently supports this pattern by showing that stress_score causes the largest ablation decline with ΔAUC = −0.114, followed by work_interfere_enc and care_options_enc, each with ΔAUC = −0.020. These findings are consistent with occupational mental-health theory because stress burden, work interference and care access are expected to shape perceived need for support.

Recent related studies also emphasize interpretability, although most rely mainly on post hoc feature attribution without latent behavioural profiling. Hasan et al. used LIME and SHAP to explain occupational stress predictions, which supports the value of local and global explanation methods in this domain [71]. Jeong et al. used SHAP to identify job instability and lack of reward as major burnout predictors, which aligns with the importance of occupational structure in mental-health risk prediction [72]. Mokheleli et al. used SHAP and LIME to interpret OSMI workplace mental-health predictions, where treatment and mental-disorder history variables were identified as influential predictors [34].

The present study extends this interpretability layer through Table 12 and Figure 8, which show two latent occupational–behavioural profiles with different support and stress patterns. Cluster 1 has higher average risk than Cluster 0 with risk values of 0.487 versus 0.448, suggesting graded workplace risk configurations rather than rigid diagnostic categories. This profiling component is useful for organizational interpretation because a probability score may indicate elevated risk, while latent profiles help explain whether that risk is associated mainly with work interference, limited support, care-access barriers or related workplace conditions. For high-pressure knowledge-work environments, including academic institutions, this distinction matters because preventive responses may require organizational changes rather than only individual recommendations.

**Table 17:** Comparison with recent occupational stress, burnout and OSMI-based mental-health prediction studies.

Study	Data/Outcome	Model	Result	Responsible-AI Elements	Comparison with Proposed Study
Hasan et al. [71]	Workplace stress survey	Ensemble ML, 1D-CNN, LLM	Accuracy = 90.32%; unseen = 89%	LIME, SHAP, ablation	Stress-focused but no RI bands or transfer testing
Jeong et al. [72]	KOSS burnout data; 1205 workers	Five ML models	RF ROC-AUC = 0.904	SHAP, stressor thresholds	Strong burnout benchmark but no latent profiling or transfer validation
Iwasaki et al. [73]	Job-stress data; LTSA risk	ML with sampling	AP = 0.040; ROC-AUC = 0.81	Imbalance handling, longitudinal validation	Shows rare-event difficulty; lacks RI and subgroup calibration
Mokheleli et al. [34]	OSMI workplace mentalhealth data	RF, XGBoost, SVM, AdaBoost	XGBoost accuracy = 91%; ROC-AUC = 90%	SHAP, LIME, SMOTE	Closest OSMI comparator; no RI, VAE or transfer evaluation
Younis [74]	OSMI; treatment willingness	RF, GB, SVM, KNN, LR	Best accuracy = 0.83	ML comparison	Same OSMI source but no calibration, fairness or external validation
Priyanka et al. [75]	OSMI Mental Health in Tech surveys; 2016–2023	Multi-scale 1D-CNN, channel-wise attention, XGBoost ensemble	Accuracy = 91.54%; F1 = 92%; precision = 92%; recall = 91%	SHAP, attention-based feature learning, local/global interpretability	Strong deep-XAI comparator; lacks RI bands, VAE latent profiling, calibration and transfer validation
Chen et al. [76]	OSMI 2014; work-interfering mental-health risk; 1259 records	LR, DT, RF, Gradient Boosting	RF recall = 0.878; F1 = 0.803; accuracy = 0.737	Gender fairness audit, feature analysis, recall-focused evaluation	Same OSMI source and fairness-oriented, but no RI bands, calibration, VAE profiling or external validation
Hendrick [77]	OSMI; mental-health disorder classification	RF, XGBoost	Accuracy gain: RF = 1.67%; XGBoost = 0.67%	Expert feature selection	Same OSMI source; no RI bands, VAE profiles or transfer testing
Mokheleli [78]	OSMI; age-stratified risk prediction	RF, XGBoost, SVM	RF/XGBoost accuracy = 91%; F1 = 93%	SHAP, age-stratified analysis	Strong subgroup interpretation; lacks calibration and cross-dataset validation
Othman and Rosdi [79]	OSMI 2014; treatment seeking	Decision Tree, KNN	DT accuracy = 73%; KNN = 100%	Interpretable classifiers	Same OSMI target; limited responsible-AI and transfer evaluation
Proposed study	OSMI plus HackerEarth burnout validation	Attention-VAE-ensemble RI model	AUC = 0.885; AP = 0.872; ECE = 0.022; external AUC = 0.941	Calibration, attention, VAE, fairness, transfer	Unified prediction, calibration, profiling, fairness and adaptation framework

Note: RI = risk index; OSMI = Open Sourcing Mental Illness; AP = average precision; ECE = expected calibration error; LTSA = long-term sickness absence; VAE = variational autoencoder; RF = random forest; GB = gradient boosting; LR = logistic regression.

### 5.3. Fairness, Transferability and External Generalization

The fairness and transfer results show that strong aggregate performance does not remove the need for subgroup-specific validation. Table 10 reports the smallest fairness gap for the proposed model with fairness gap = 0.169, compared with 0.184 for CatBoost, 0.181 for FT–Transformer and 0.206 for random forest. Table 14 also shows country-level subgroup variation with AUC = 0.903 for Germany, 0.880 for the United Kingdom, 0.868 for the United States and 0.734 for Canada. These differences indicate that aggregate model quality can conceal regional reliability gaps, especially when subgroup sizes, reporting norms, care-access conditions and occupational contexts differ.

Transfer learning further confirms the importance of adaptation before deployment because regional portability remains uneven across available partitions. Table 15 shows that United States models transfer better than several smaller sources with self AUC = 0.698 and mean cross-region AUC = 0.637. By contrast, the United Kingdom has self AUC = 0.465 and mean cross-region AUC = 0.492, which suggests weak portability under the available regional partition. This concern is consistent with Iwasaki et al., who found that predicting rare long-term sickness absence due to mental disorders remained difficult even with a large occupational cohort, reporting AP = 0.040 and ROC-AUC = 0.81 [73].

Table 16 provides an additional external test using the HackerEarth burnout dataset. Direct source-only transfer from OSMI is weak with AUC = 0.642 and ECE = 0.128, which confirms substantial dataset shift. In contrast, the final harmonized and adapted model reaches AUC = 0.941, AP = 0.936, ECE = 0.029 and fairness gap = 0.078, showing that cross-dataset occupational risk modelling becomes more reliable when conceptual harmonization, target labels, recalibration and adaptation are used together. These findings should not be interpreted as evidence that an OSMI-trained model can be deployed directly in any occupational or academic setting without local validation.

Overall, the discussion supports three connected conclusions about calibrated occupational mental-health risk modelling. First, calibrated risk indexing improves practical interpretability by converting probabilities into operational risk bands for support prioritization. Second, attention analysis, perturbation testing and latent profiling provide convergent evidence that the model relies on plausible occupational signals, especially stress burden, work interference and care-access variables. Third, subgroup and transfer results show that responsible use requires local validation, recalibration and fairness monitoring before deployment in new workplace settings. These requirements are especially important if the framework is later applied to early-career academics or other high-pressure knowledge-work populations because the present empirical datasets represent broader workplace cohorts rather than direct ECR-only samples.

## 6. Conclusions and Future Work

In conclusion, this study developed an interpretable deep-learning framework for occupational mental-health risk modelling using heterogeneous workplace survey data. The proposed approach combined attention-based tabular learning, calibrated probability mapping, latent occupational–behavioural profiling, ensemble prediction, fairness assessment and transfer validation within a single computational pipeline. The framework was motivated by the need for risk-modelling tools that are relevant to high-pressure knowledge-work populations, including early-career academic communities, while the empirical development and validation were conducted on broader workplace mental-health and burnout datasets.

Quantitatively, the proposed attention-VAE-ensemble risk-index model achieved strong held-out performance with AUC = 0.885, average precision = 0.872 and F1 = 0.808, outperforming logistic regression, support vector machine, random forest, XGBoost, LightGBM, CatBoost, TabNet, FT–Transformer, NODE and DCN under the same evaluation protocol. The model also produced reliable calibrated risk estimates with Brier score = 0.145 and expected calibration error = 0.022, supporting the conversion of calibrated probabilities into operational 0–100 risk-index scores. Robustness analysis showed a conservative five-fold mean test AUC of 0.809±0.044, indicating moderate sensitivity to data partitioning but stable predictive utility across repeated validation. External cross-dataset evaluation on the occupational burnout cohort further demonstrated practical generalization after harmonization, target-domain adaptation and recalibration, achieving AUC = 0.941 and average precision = 0.936. These results indicate that the framework is effective not only for discrimination but also for calibrated risk stratification, subgroup-aware interpretation and transfer-oriented occupational mental-health decision support. Key predictors such as work_interfere and stress_score were consistently identified across attention, ablation and perturbation analyses, while latent profiling revealed distinct occupational–behavioural subgroups with different support, stress and work-interference patterns.

The findings also highlight important limitations that should guide future development. Subgroup and transfer analyses showed that model performance can vary across countries and demographic groups, indicating that aggregate accuracy is insufficient for responsible institutional deployment. Fairness gaps, subgroup calibration differences and weaker transfer in smaller or more heterogeneous regions suggest that future implementations should include local validation, subgroup-aware calibration and bias-mitigation procedures before practical use. The cross-dataset evaluation further showed that direct source-only transfer is unreliable under dataset shift, whereas harmonization, target-domain labels, recalibration and adaptation substantially improve external performance. Synthetic data experiments also showed mixed fidelity, especially for continuous variables, meaning that generated records should remain restricted to robustness checks unless stronger distributional validation is achieved.

Future work should extend the framework using longitudinal academic workforce data to capture temporal changes in stress, support availability, workload pressure, mentoring access and help-seeking behaviour. Such data would allow the framework to be tested directly in early-career academic populations rather than inferred from broader workplace cohorts. Additional work should examine domain-adaptation methods, fairness-aware thresholding and causal validation strategies to improve reliability across institutions, countries and career stages. Integrating multimodal information, including workload logs organizational policy indicators, mentoring availability and repeated well-being assessments, may further strengthen predictive accuracy while preserving interpretability. From a practical perspective, the reported AUC, average precision, calibration error and external-validation results suggest that the proposed framework can support preventive outreach prioritization, provided that local validation, recalibration and fairness monitoring are conducted before deployment.

## Figures and Tables

**Table 1:** Comparison of related computational approaches for occupational and public mental health risk modelling.

Study Type	Occupational/ECR Relevance	Tabular Health Data	Attention-Based Modelling	VAE/Latent Profiling	Calibration	Interpretability	Fairness/Subgroup Evaluation	Transfer Validation
Public AI [38,67]	×	✓	Δ	×	Δ	Δ	Δ	×
Academic MH [40,68,69]	✓	Δ	×	×	×	Δ	Δ	×
General ML classifiers [42]	Δ	✓	×	×	Δ	Δ	Δ	×
Deep Tabular [46,52,70]	×	✓	✓	×	Δ	Δ	×	Δ
Attention-based deep learning [16,48,49]	Δ	✓	✓	×	Δ	Δ	×	Δ
Responsible AI [58,59,64,65,66]	×	✓	Δ	×	✓	✓	✓	✓
Our study	Δ	✓	✓	✓	✓	✓	✓	✓

Note: ✓ = fully addressed; Δ = partially addressed or addressed as a motivating/application context; × = not addressed or not central to the study. ECR = early-career researcher; VAE = variational autoencoder; MH = mental health; ML = machine learning.

**Table 2:** Dataset summary and analytical role.

Attribute	Description
Dataset name	Mental Health in Tech Survey (Open Sourcing Mental Illness)
Sample	Working respondents from a workplace mental-health survey, used as a development cohort for occupational risk modelling
Sample size	Around 1000–2000 respondents after filtering and cleaning
Coverage	Multi-country sample, mainly North America and Europe
Inputs	Demographics, workplace factors, support, stigma, benefits, access to care and help-seeking attitudes
Outcome	Binary indicator of likely need for mental health support
Analytical role	Suitable for attention-based modelling, calibration, latent profiling, fairness checks and transfer evaluation
Access	Public dataset, subject to repository and license terms

**Table 3:** Preprocessing, feature engineering and training protocol.

Component	Operation	Implementation	Purpose
Missingness	Median fill with masks	Training medians and binary indicators	Preserve missingness without leakage
Scaling	Standardization	Training-set mean and standard deviation	Align numerical inputs
Categorical encoding	Integer indexing and embeddings	Trainable embedding tables	Represent sparse categories
Feature filtering	Variance and correlation screening	Low-variance and redundancy filtering	Remove unstable predictors
Composite features	Workload, support, barrier and access summaries	Weighted survey-item aggregation	Capture psychosocial constructs
Class imbalance	Stratified splits and weighted focal loss	No oversampling in main training	Preserve empirical distribution
Optimizer	AdamW with Adam checks	Warmup and cosine decay	Stabilize convergence
Batch and regularization	Batch size 64–128, dropout, weight decay	Hardware-dependent selection	Reduce overfitting
Early stopping	Validation-loss monitoring	Patience and best-weight restoration	Prevent late-epoch overfitting

**Table 4:** Evaluation, latent validation, fairness assessment and transfer protocol.

Evaluation Domain	Metrics	Procedure	Purpose
Discrimination	AUC and average precision	Stratified test split and cross-validation	Evaluate ranking and minority retrieval
Calibration	Brier score and expected calibration error	Probability-bin comparison and validation calibration	Assess probability reliability
Statistical testing	Bootstrap CI and DeLong test	Non-parametric resampling for metric uncertainty and paired ROC-AUC comparison for model discrimination	Quantify confidence intervals and statistical significance of performance differences
Latent validity	Silhouette score and Davies–Bouldin index	Multi-seed VAE and GMM evaluation	Test cluster stability
Synthetic validity	KS distance and EMD	Real-versus-synthetic comparison	Detect distribution mismatch
Interpretability stability	SHAP rank agreement and perturbation AUC drop	Attribution and masking tests	Evaluate explanation robustness
Fairness	Subgroup AUC, TPR gap, TNR gap and calibration gap	Region and demographic subgroup analysis	Identify uneven errors
Transfer learning	Source-to-target fine-tuning and AUC drop	Train source, fine-tune target	Assess adaptation need
Baseline comparison	Pooled global model and target-only model	Matched regional protocol	Benchmark transfer results

**Table 5:** Hyperparameter selection and reproducibility controls.

Component	Parameter or Control	Selected Value	Role
TabTransformer	Embedding dimension	32	Dense categorical representation
TabTransformer	Attention heads	4	Multi-context interaction learning
TabTransformer	Feedforward width	256	Nonlinear transformation
TabNet	Decision steps	5	Feature-selection depth
TabNet	Transformer dimension	64	Representation width
VAE	Latent dimension	16	Behavioural profile representation
VAE	KL weight β	0.5	Reconstruction and regularization balance
Ensemble	Meta-learner units	64	Prediction fusion capacity
Ensemble	Weight regularization	$1\times 10^{-3}$	Overfitting control
Calibration	Platt scaling	Learned on validation data	Probability reliability
Randomness	Seed control	Fixed seed across splits and sampling	Reproducible comparison
Determinism	Backend settings	Deterministic operations where available	Stable execution
Versioning	Package and environment records	Locked versions with run logs	Environment replication
Hardware	GPU and CPU logging	Recorded per experiment	Performance traceability

**Table 6:** State-of-the-art baseline comparison protocol.

Baseline Family	Models	Tuned Parameters	Purpose
Linear and probabilistic models	Logistic regression, ridge logistic model	Regularization strength, class weight	Test calibrated linear structure
Kernel and margin models	Support vector machine	Kernel type, penalty, class weight	Test nonlinear margins
Tree ensembles	Random forest, extra trees	Trees, depth, split size, class weight	Provide non-neural tabular baselines
Gradient boosting	XGBoost, LightGBM, CatBoost	Learning rate, depth, estimators, leaf size, regularization	Compare against tabular SOTA methods
Deep tabular models	TabNet, FT–Transformer, NODE, DCN	Embedding size, attention heads, decision steps, hidden width	Compare neural representation learning
Calibrated variants	Platt-scaled and isotonic-calibrated baselines	Calibration method, validation split	Compare probability reliability
Proposed framework	Attention-VAE-ensemble RI model	Settings from Table 5	Evaluate integrated prediction, calibration, profiling and fairness validation

**Table 7:** Cross-dataset evaluation protocol using the HackerEarth Employee Burnout Challenge dataset.

Evaluation Setting	Training Data	Testing Data	Purpose
Source-only transfer	OSMI survey only	HackerEarth burnout dataset	Assess external generalization
Target-only baseline	HackerEarth training split only	HackerEarth test split	Estimate local performance
Pooled learning	OSMI plus HackerEarth harmonized training data	HackerEarth test split	Test shared occupational-risk signals
Target-adapted fine-tuning	OSMI model plus small HackerEarth labelled subset	HackerEarth test split	Assess adaptation with limited labels
Feature-harmonized model	Conceptually aligned variables only	OSMI and HackerEarth test splits	Ensure defensible comparison
Burnout-label conversion	HackerEarth Burn Rate converted to binary high-risk label	HackerEarth test split	Align with risk classification
Calibration transfer	Source calibration and target recalibration	HackerEarth validation and test splits	Evaluate probability reliability
Fairness check	Shared subgroup variables such as gender and work arrangement	HackerEarth test split	Examine subgroup reliability

**Table 8:** Hardware and software environment used for model implementation and evaluation.

Component	Configuration
Operating system	Ubuntu 22.04 LTS, 64-bit
Programming language	Python 3.10.14
Processor	x86-64 workstation CPU
System memory	32 GB RAM
Graphics processor	NVIDIA GeForce RTX 3060
GPU memory	12 GB VRAM
Deep-learning framework	PyTorch 2.2.2
Machine-learning libraries	Scikit-learn 1.4.2, XGBoost 2.0.3, LightGBM 4.3.0, CatBoost 1.2.5
Data-processing libraries	NumPy 1.26.4, Pandas 2.2.2
Interpretability tools	SHAP 0.45.1, integrated gradients and perturbation attribution
Calibration and evaluation	ROC-AUC, average precision, F1 score, Brier score and ECE
Reproducibility controls	Fixed seeds, stratified splits, deterministic settings and experiment logs

**Table 9:** Calibration summary and risk-index mapping across probability bands and operational score levels.

Calibration by Probability Range			
Prob Range	Mean Predicted	Fraction Positive	Count
0.00 to 0.20	0.069575	0.076923	78
0.20 to 0.50	0.358531	0.422222	45
0.50 to 0.80	0.688924	0.693877	49
0.80 to 1.00	0.886547	0.862500	80
Overall Discriminative and Calibration Metrics			
AUC ROC	Average Precision AP	Brier Score	ECE
0.88199	0.86884	0.145	0.022
Risk Index Mapping			
Label	Prob Range	Score Range	Note
Very low	0.00 to 0.25	1.6 to 11.2	Conservative low-risk band
Low	0.25 to 0.50	11.2 to 55.4	Increased monitoring
Moderate	0.50 to 0.75	55.4 to 85.8	Outreach recommended
High	0.75 to 1.00	85.8 to 96.3	Prioritized outreach and assessment

**Table 10:** Comparison with state-of-the-art baseline models across discrimination, calibration and fairness metrics.

Model	AUC	AP	F1	Brier	ECE	Fairness Gap
Logistic regression	0.829	0.795	0.724	0.165	0.034	0.176
Support vector machine	0.841	0.807	0.736	0.177	0.051	0.189
Random forest	0.882	0.859	0.802	0.153	0.039	0.206
XGBoost	0.876	0.852	0.790	0.151	0.034	0.195
LightGBM	0.879	0.856	0.797	0.149	0.032	0.190
CatBoost	0.881	0.860	0.801	0.148	0.030	0.184
TabNet	0.868	0.840	0.782	0.158	0.044	0.193
FT–Transformer	0.874	0.848	0.791	0.154	0.037	0.181
NODE	0.862	0.833	0.774	0.160	0.046	0.197
DCN	0.858	0.827	0.768	0.163	0.048	0.202
Proposed attention-VAE-ensemble RI model	0.885	0.872	0.808	0.145	0.022	0.169

**Table 11:** Feature interactions, perturbation sensitivity and five-fold cross-validation robustness analysis.

Top Feature Interactions				
Feat i	Feat j	Interaction	*p*-Value	Interpretation
work_int	anon	0.009	0.139	Joint positive effect
gender	remote	0.009	0.020	Significant joint effect
country	mh_intervw	0.004	0.020	Demographic-attitude link
work_int	mh_consq	−0.009	0.931	Weak negative association
Perturbation Impact on AUC				
Feature	Base AUC	Perturbed AUC	ΔAUC	Interpretation
work_int	0.844	0.657	−22.1%	Critical performance drop
stress	0.844	0.843	−0.1%	Negligible effect
support	0.844	0.842	−0.2%	Robust to perturbation
country	0.844	0.846	+0.3%	Mild sensitivity
anon	0.844	0.846	+0.3%	Possible redundancy
Cross-Validation Robustness Analysis, 5-fold				
Metric	Mean	Std	Min	Max
Val AUC	0.846	0.019	0.826	0.873
Val AP	0.794	0.035	0.756	0.834
Test AUC	0.809	0.044	0.734	0.844
Test F1	0.746	0.057	0.678	0.808

**Table 12:** Cluster centroids showing averaged categorical modes across two behavioural profiles.

Feature	$C_{0}$	$C_{1}$	Feature	$C_{0}$	$C_{1}$
Country	US	US	seek_help	Yes	Yes
state	CA	NY	anonymity	DK	DK
self_emp.	No	No	leave	Somewhat	Somewhat
fam_hist	No	No	mh_consq	Maybe	Maybe
work_int	Rarely	Often	ph_consq	No	Maybe
employees	1–5	26–100	coworkers	Yes	Some
remote	Yes	Yes	supervisor	Yes	No
tech_co	Yes	Yes	mh_intervw	No	No
benefits	No	No	ph_intervw	Maybe	No
care_opt	No	No	mh_vs_ph	Yes	Yes
wellness	No	No	obs_consq	No	No

**Table 13:** Evaluation summary covering downstream performance, latent clusters and synthetic validity checks.

Downstream Performance			
Model	AUC	AP	Metrics
RF baseline	0.882	0.859	0.816/0.802/0.771/0.867
RF augmented	0.879	0.864	0.810/0.794/0.760/0.867
Cluster Summaries			
Cluster	Size	Signature	Avg risk
0	649	↑ support/↓ stress	0.448
1	610	↓ support/↑ stress	0.487
Synthetic vs. real			
Feature	KS	EMD	Verdict
Age	0.778	$1.2\times 10^{8}$	Divergent
stress_score	0.589	0.530	Divergent
support_score	0.383	0.462	Acceptable
Key categoricals	small	0.4–0.6	Reasonable

Note: ↑ indicates a higher relative feature tendency within the cluster, whereas ↓ indicates a lower relative feature tendency.

**Table 14:** Ablation and subgroup fairness summary across influential features and regional groups.

Top Ablations (ΔAUC)		Selected Subgroup AUC	
Feature	ΔAUC	Subgroup	AUC
stress_score	−0.114	United States	0.868
work_interfere_enc	−0.020	United Kingdom	0.880
care_options_enc	−0.020	Germany	0.903
benefits_enc	−0.007	Canada	0.734

**Table 15:** Transfer learning performance across regions using self-transfer and cross-region AUC summaries.

Region	Self AUC	Mean Cross AUC	Baseline AUC
United States	0.698	0.637	0.449
United Kingdom	0.465	0.492	0.510
Canada	0.537	0.532	0.648
Germany	0.677	0.579	0.670
Netherlands	0.594	0.540	0.519
Ireland	0.600	0.531	0.538

**Table 16:** Cross-dataset evaluation results using the HackerEarth Employee Burnout Challenge dataset.

Training Setting	Development Data	External Test Data	AUC	AP	ECE	Fairness Gap
Source-only transfer	OSMI survey	HackerEarth burnout dataset	0.642	0.617	0.128	0.214
Target-only baseline	HackerEarth training split	HackerEarth test split	0.932	0.925	0.041	0.092
Pooled learning	OSMI plus HackerEarth harmonized training data	HackerEarth test split	0.919	0.913	0.049	0.104
Target-adapted fine-tuning	OSMI model plus HackerEarth labelled subset	HackerEarth test split	0.935	0.929	0.038	0.086
Source calibration only	OSMI calibrated model	HackerEarth test split	0.642	0.617	0.132	0.214
Target recalibration	OSMI model recalibrated on HackerEarth validation data	HackerEarth test split	0.642	0.617	0.052	0.208
Proposed final cross- dataset model	Harmonized and adapted OSMI-HackerEarth model	HackerEarth test split	0.941	0.936	0.029	0.078

## Data Availability

The datasets used in this study are publicly available on Kaggle. Dataset A is the OSMI Mental Health in Tech Survey, available at Dataset A Link: https://www.kaggle.com/datasets/osmi/mental-health-in-tech-survey (accessed on 24 April 2026). Dataset B is the HackerEarth Employee Burnout Challenge dataset, available at Dataset B Link: https://www.kaggle.com/datasets/redwankarimsony/hackerearth-employee-burnout-challenge (accessed on 24 April 2026). All code used for data preprocessing, model training, evaluation and figure generation is available at Code Link: https://github.com/abuzarkhaaan/Deep-Learning-Based-Predictive-Modeling- (accessed on 15 November 2025). The repository includes configuration files and scripts to reproduce the main results reported in this paper.

## References

[1] Herstad S.J. (2026). Knowledge work and occupational stress. Ind. Innov..

[2] Hammoudi Halat D., Soltani A., Dalli R., Alsarraj L., Malki A. (2023). Understanding and Fostering Mental Health and Well-Being among University Faculty: A Narrative Review. J. Clin. Med..

[3] van der Weijden I., Teelken C. (2023). Precarious careers: Postdoctoral researchers and wellbeing at work. Stud. High. Educ..

[4] Choi M.H., Choi J.S. (2024). Comparing Brain Activation Patterns in Stress-Induced and Post-Stress Recovery States of Highly and Moderately Stressed Individuals. Appl. Sci..

[5] Kelloway E.K., Dimoff J.K., Gilbert S. (2023). Mental Health in the Workplace. Annu. Rev. Organ. Psychol. Organ. Behav..

[6] Khan A., Masood F., Iqbal A., Junaid A., Arif S., Al-Naeem M., Husnain G., Alzahrani A.S. (2026). Evaluating Routing Stability and Coordination in Swarm-Based Multi-Agent Task-Oriented Dialogue Systems. Sci. Rep..

[7] Zile A., Porter B., Crozier K., Sanderson K. (2023). The Mental Health of UK Postgraduate Research Students following the COVID-19 Pandemic. Educ. Sci..

[8] Colecchia F., Spinelli G., Havsteen-Franklin D., Nandy M. (2025). Toward a Sociotechnical Ecosystem for Ethical Screening and Promotion of Mental Health and Well-Being. J. Med. Internet Res..

[9] Khan A., Junaid A., Husnain G., Alzahrani K.J., Alkahtani H.K. (2026). An Efficient Intrusion Detection System Using Domain-Aware Meta-Learning with Adapter-Based Few-Shot Adaptation in Vehicular Ad-Hoc Networks (VANETs). IET Intell. Transp. Syst..

[10] Fei Z., Ryeznik Y., Sverdlov O., Tan C.W., Wong W.K. (2022). An Overview of Healthcare Data Analytics With Applications to the COVID-19 Pandemic. IEEE Trans. Big Data.

[11] Kerr J.I., Naegelin M., Weibel R.P., Ferrario A., La Marca R., von Wangenheim F., Hoelscher C., Schinazi V.R. (2020). The effects of acute work stress and appraisal on psychobiological stress responses in a group office environment. Psychoneuroendocrinology.

[12] Khan A., Junaid A., Iqbal A., Iqbal S., Almuqren L., Husnain G., Bukhari S.H.R., Al-Naeem M. (2026). Smart Sensing-Enabled Risk-Aware Nitrogen Prescriptions via Conformal Profit Bounds for Precision Agriculture. Front. Plant Sci..

[13] Iwamoto H., Nakano S., Tajima R., Kiguchi R., Yoshida Y., Kitanishi Y., Aoki Y. (2024). Predicting Workers’ Stress: Application of a High-Performance Algorithm Using Working-Style Characteristics. JMIR AI.

[14] Liu J., Tian T., Liu Y., Hu S., Li M. (2023). iTabNet: An improved neural network for tabular data and its application to predict socioeconomic and environmental attributes. Neural Comput. Appl..

[15] Isomura T., Shimizu R., Goto M. (2025). Sparse attention is all you need for pre-training on tabular data. Neural Comput. Appl..

[16] Li H., Dong S. (2024). Image Steganalysis Algorithm Based on Deep Learning and Attention Mechanism for Computer Communication. J. Electron. Imaging.

[17] Wang N., Wu M., Gu W., Dai C., Shao Z., Subbalakshmi K.P. (2025). MSFT-transformer: A multistage fusion tabular transformer for disease prediction using metagenomic data. Brief. Bioinform..

[18] Atzmueller M., Fürnkranz J., Kliegr T., Schmid U. (2024). Explainable and interpretable machine learning and data mining. Data Min. Knowl. Discov..

[19] Junaid A., Iqbal A., Khan A., Husnain G., Ahmad A.R., Al-Naeem M. (2026). Engine Failure Prediction on Large-Scale CMAPSS Data Using Hybrid Feature Selection and Imbalance-Aware Learning. Comput. Mater. Contin..

[20] Hang C.N., Yu P.D., Chen S., Tan C.W., Chen G. (2023). MEGA: Machine Learning-Enhanced Graph Analytics for Infodemic Risk Management. IEEE J. Biomed. Health Inform..

[21] Cui C., Liu L., Qiao R. (2024). A Cutting-Edge Video Anomaly Detection Method Using Image Quality Assessment and Attention Mechanism-Based Deep Learning. Alex. Eng. J..

[22] Zhang S., Han Q., Wang P., Li J. (2025). Frame Topology Fusion-Based Hierarchical Graph Convolution for Automatic Assessment of Physical Rehabilitation Exercises. Sci. Rep..

[23] Abbas K., Hasan M.K., Abbasi A., Mokhtar U.A., Khan A., Abdullah S.N.H.S., Dong S., Islam S., Alboaneen D., Ahmed F.R.A. (2023). Predicting the Future Popularity of Academic Publications Using Deep Learning by Considering It as Temporal Citation Networks. IEEE Access.

[24] Bin-Salem A.A., Zubaydi H.D., Alzubaidi M., Tariq Z.U.A., Naeem H. (2022). A Scoping Review on COVID-19’s Early Detection Using Deep Learning Model and Computed Tomography and Ultrasound. Trait. Du Signal.

[25] Zhang C., Guo Z., Li C. (2025). Unsupervised Anomaly Detection for Gearboxes Based on the Deep Convolutional Support Generative Adversarial Network. Sci. Rep..

[26] Husnain G., Ali A.B.M., Khan A., Junaid A., Usman M., Awwad E.M. (2026). ViT-Xplain: A Transparent Deepfake Detector for Consumer Electronics Based on Attention and Explainable AI. IEEE Trans. Consum. Electron..

[27] Liu L., Chu C., Chen C., Huang S. (2024). MarineYOLO: Innovative Deep Learning Method for Small Target Detection in Underwater Environments. Alex. Eng. J..

[28] Yin X., Chen L. (2024). Image Object Detection Method Based on Improved Faster R-CNN. J. Circuits Syst. Comput..

[29] Wang Y., Feng Y., Sun H. (2021). Research on Vehicle Intelligent Wireless Location Algorithm Based on Convolutional Neural Network. Neural Comput. Appl..

[30] Fan J., Yu G.A., Zhao M., Zong H. (2025). Addressing Multi-Scale Temporal Variability: Deep Integration and Application of the CNN and Transformer Model in Monthly Streamflow Prediction. Expert Syst. Appl..

[31] Qi H.X., Yang S.Y., Miao Y.H., Cui L.G., Xue H., Hua J.D., Hong K., Fang Y.Y. (2026). Prediction of Sperm Retrieval Outcomes Based on Testicular Ultrasound Images and Dense Convolutional Sparse Coding. IEEE Sens. J..

[32] Meng L., Xi X., Han J., Qiao L., Yin Y., Chen X. (2026). Difficulty-aware pseudo-label correction network for fine-grained classification of choroidal neovascularization in OCT images. IEEE Trans. Multimed..

[33] Chen P., Nie X., Ning Y., Zhang Y. (2025). Learning Efficient and Adaptive Cross-Channel Dependencies for Weakly-Supervised Object Detection. IEEE Trans. Multimed..

[34] Mokheleli T., Bokaba T., Mbunge E. (2025). Explainable Artificial Intelligence for Workplace Mental Health Prediction. Informatics.

[35] Wang L., Bala H., Yan L., Guo X. (2026). Physicians’ Contributions to Online Healthcare Platforms: Relative Effects of Herding Cues and Feedback Types. J. Manag. Inf. Syst..

[36] Wang L., Ma Y., Yan Z., Zhang L., Hu Y., Zhao S. (2025). Giving or Receiving: Impact of Online Socializing in Online Fitness Community on Physical Activity and Emotional State. Comput. Hum. Behav..

[37] Zheng X., Yu H., Cui H., Sun C., Li X., Su R., Wei L., Zhou J., Wang J., Jin Q. (2026). KG-CMI: Knowledge Graph Enhanced Cross-Mamba Interaction for Medical Visual Question Answering. IEEE Trans. Ind. Inform..

[38] Ali M., Ali S., Abbas Q., Abbas Z., Lee S.W. (2025). Artificial intelligence for mental health: A narrative review of applications, challenges, and future directions in digital health. Digit. Health.

[39] Khan A., Junaid A., Husnain G., Algarni A., Al-Rasheed A., Mostafa H.A. (2026). Risk-Aware Federated Hierarchical Reinforcement Learning for Cooperative CAV Safety in Intelligent Trans1:09 PM 5/14/2026portation System. IET Intell. Transp. Syst..

[40] Di Giacomo D., Cilli E., Ranieri J., Guerra F., Martelli A. (2024). Mental health of young researchers in academia: Towards to growth perspective. Pers. Med. Psychiatry.

[41] Khan A., Junaid A., Siddique M.F., Iqbal A., Samkari H.S., Allehyani M.F., Husnain G. (2026). Smart Predictive Maintenance: A TCN-Based System for Early Fault Detection in Industrial Machinery. Machines.

[42] Dong S. (2021). Multi Class SVM Algorithm with Active Learning for Network Traffic Classification. Expert Syst. Appl..

[43] Iyortsuun N.K., Kim S.H., Jhon M., Yang H.J., Pant S. (2023). A Review of Machine Learning and Deep Learning Approaches on Mental Health Diagnosis. Healthcare.

[44] Khan A., Iqbal A., Husnain G., Masood F., Al-Naeem M., Iqbal S. (2026). Secure and Differentially Private Edge-Cloud Federated Learning Framework for Privacy-Preserving Maritime AIS Intelligence. Comput. Mater. Contin..

[45] Husnain G., Zafar W., Iqbal A., Khan A., Alzahrani A.S., Al-Naeem M. (2026). A Biologically Inspired Intelligent and Energy Efficient Route Optimization Clustering Algorithm for Internet of Vehicles (IoV). IET Intell. Transp. Syst..

[46] Madan S., Lentzen M., Brandt J., Rueckert D., Hofmann-Apitius M., Fröhlich H. (2024). Transformer models in biomedicine. BMC Med. Inform. Decis. Mak..

[47] Khan A., Al Farid F., Junaid A., Siddique M.F., Iqbal A., Siddique M.S., Uddin J., Karim H.A., Husnain G. (2026). Early-warning industrial fault detection based on physics-guided residual learning and calibrated CRNNs. Sci. Rep..

[48] Kang H.Y.J., Ko M., Ryu K.S. (2025). Tabular transformer generative adversarial network for heterogeneous distribution in healthcare. Sci. Rep..

[49] Chen J., Zhao W., Cui Y., Wei C., Polat K., Alenezi F. (2026). A review of EEG-based driver fatigue detection: Nonlinear dynamics, brain networks, and deep learning advances. WIREs Data Min. Knowl. Discov..

[50] Chen J., Jin S., Cui Y., Wei C., Polat K., Alenezi F. (2026). Entropy-Informed Deep Residual Network for Nonlinear EEG Dynamics in Fine-Grained Driver State Recognition. Appl. Soft Comput..

[51] Yuan Q., Sun W., Li F., Dong X., Yuan Y. (2026). A dual attention transformer modelling for explainable mental health analysis in academic environments using TaBERT. Sci. Rep..

[52] Lee Y.H., Lee J.H., Auh Q.S., Lee S., Nixdorf D., Chaurasia A. (2026). TMD Diagnosis Using a Masked Self-Supervised Tabular Transformer. J. Dent. Res..

[53] Murtaza H., Ahmed M., Khan N.F., Murtaza G., Zafar S., Bano A. (2023). Synthetic data generation: State of the art in health care domain. Comput. Sci. Rev..

[54] Gao G., Chen C., Xu K., Liu K., Mashhadi A. (2024). Automatic Face Detection Based on Bidirectional Recurrent Neural Network Optimized by Improved Ebola Optimization Search Algorithm. Sci. Rep..

[55] Dong S., Shu L., Nie S. (2024). Android Malware Detection Method Based on CNN and DNN Bybrid Mechanism. IEEE Trans. Ind. Inform..

[56] Naeem H., Cheng X., Ullah F., Jabbar S., Dong S. (2022). A Deep Convolutional Neural Network Stacked Ensemble for Malware Threat Classification in Internet of Things. J. Circuits Syst. Comput..

[57] Naeem H., Alsirhani A., Alshahrani M.M., Alomari A. (2022). Android Device Malware Classification Framework Using Multistep Image Feature Extraction and Multihead Deep Neural Ensemble. Trait. Signal.

[58] Collins G.S., Moons K.G.M., Dhiman P., Riley R.D., Beam A.L., Van Calster B., Ghassemi M., Liu X., Reitsma J.B., van Smeden M. (2024). TRIPOD + AI statement: Updated guidance for reporting clinical prediction models that use regression or machine learning methods. BMJ.

[59] Efthimiou O., Seo M., Chalkou K., Debray T., Egger M., Salanti G. (2024). Developing clinical prediction models: A step-by-step guide. BMJ.

[60] Li H., Li Y., Li P., Zhang G., Wang W., Xu K. (2025). Exploring Uncertainty and Representativeness for Deep Active Learning. J. Circuits Syst. Comput..

[61] Dong S., Sarem M. (2020). DDoS Attack Detection Method Based on Improved KNN With the Degree of DDoS Attack in Software-Defined Networks. IEEE Access.

[62] Wang H., Zhao L., Peng Q. (2025). An Improved Sand Cat Swarm Optimization Algorithm and Its Application to Agricultural Robot Path Planning. Eng. Comput..

[63] Zhang D.L., Jiang Z., Mohammadzadeh F., Hasani Azhdari S.M., Abualigah L., Ghazal T.M. (2024). FUZ-SMO: A Fuzzy Slime Mould Optimizer for Mitigating False Alarm Rates in the Classification of Underwater Datasets Using Deep Convolutional Neural Networks. Heliyon.

[64] Xu Z., Li J., Yao Q., Li H., Zhao M., Zhou S.K. (2024). Addressing fairness issues in deep learning-based medical image analysis: A systematic review. npj Digit. Med..

[65] Naderalvojoud B., Curtin C., Asch S.M., Humphreys K., Hernandez-Boussard T. (2025). Evaluating the impact of data biases on algorithmic fairness and clinical utility of machine learning models for prolonged opioid use prediction. JAMIA Open.

[66] van der Meijden S.L., van Boekel A.M., Schinkelshoek L.J., van Goor H., Steyerberg E.W., Nelissen R.G.H.H., Mesotten D., Geerts B.F., de Boer M.G.J., Arbous M.S. (2025). Development and validation of artificial intelligence models for early detection of postoperative infections (PERISCOPE): A multicentre study using electronic health record data. Lancet Reg. Health Eur..

[67] Islam M.M., Hassan S., Akter S., Jibon F.A., Sahidullah M. (2024). A comprehensive review of predictive analytics models for mental illness using machine learning algorithms. Healthc. Anal..

[68] Rugulies R., Aust B., Greiner B.A., Arensman E., Kawakami N., LaMontagne A.D., Madsen I.E.H. (2023). Work-related causes of mental health conditions and interventions for their improvement in workplaces. Lancet.

[69] Hanitzsch T., Markiewitz A., Bødker H. (2024). Publish and perish: Mental health among communication and media scholars. J. Commun..

[70] Kannan M., Umamaheswari D., Manimekala B., Priya Stella Mary I., Margaret Savitha P., Rozario J. (2025). An enhancement of machine learning model performance in disease prediction with synthetic data generation. Sci. Rep..

[71] Hasan M.J., Sultana J., Ahmed S., Momen S. (2025). Early detection of occupational stress: Enhancing workplace safety with machine learning and large language models. PLoS ONE.

[72] Jeong H., Yang S.C., Park S.G., Hong I., Kim H.D. (2025). Predicting the Risk of Burnout Syndrome Using Korean Occupational Stress Scale (KOSS): A Machine Learning Approach. Saf. Health Work..

[73] Iwasaki S., Deguchi Y., Okura S., Maekubo K., Matsunaga A., Inoue K. (2026). Machine learning prediction of long-term sickness absence due to mental disorders using Brief Job Stress Questionnaire data. Sci. Rep..

[74] Younis M.C. (2024). Prediction of Patient’s Willingness for Treatment of Mental Illness Using Machine Learning Approaches. Appl. Comput. Sci..

[75] Priyanka, Nagpal S., Sabharwal S. (2026). MH-XAI: Hybrid Deep Learning and XGBoost Explainable AI Model for Mental Health Prediction. Concurr. Comput. Pract. Exp..

[76] Chen J., Ouyang H., Xu Y., Kong S. (2025). Mental Health In Tech Survey. Appl. Comput. Eng..

[77] Hendrick (2025). Analysis of the Impact of Interview-Based Feature Selection on the Performance of Machine Learning Algorithms in Mental Health Disorder Classification. J. Komput. Inf. Dan Teknol..

[78] Mokheleli T. (2026). Age-Stratified Mental Health Prediction Using SHAP: An Explainable Artificial Intelligence Framework. ADCAIJ Adv. Distrib. Comput. Artif. Intell. J..

[79] Othman N.A., Rosdi M. (2026). Machine Learning Approaches to Workplace Mental Health: Predicting Treatment-Seeking Behavior Using the OSMI Dataset. J. Tech-E.

